# Senescence-related impairment of autophagy induces toxic intraneuronal amyloid-β accumulation in a mouse model of amyloid pathology

**DOI:** 10.1186/s40478-023-01578-x

**Published:** 2023-05-17

**Authors:** Nuria Suelves, Shirine Saleki, Tasha Ibrahim, Debora Palomares, Sebastiaan Moonen, Marta J. Koper, Céline Vrancx, Devkee M. Vadukul, Nicolas Papadopoulos, Nikenza Viceconte, Eloïse Claude, Rik Vandenberghe, Christine A. F. von Arnim, Stefan N. Constantinescu, Dietmar Rudolf Thal, Anabelle Decottignies, Pascal Kienlen-Campard

**Affiliations:** 1 Aging and Dementia Group, Cellular and Molecular Division (CEMO), Institute of Neuroscience (IoNS), UCLouvain, Brussels, Belgium; 2 Laboratory for Neuropathology, Department of Imaging and Pathology, Leuven Brain Institute (LBI), KU Leuven, Leuven, Belgium; 3 Laboratory for the Research of Neurodegenerative Diseases, Department of Neurosciences, Leuven Brain Institute (LBI), KU Leuven, Leuven, Belgium; 4 Vlaams Instituut Voor Biotechnologie (VIB) Center for Brain and Disease Research, VIB, Leuven, Belgium; 5 Present Address: Laboratory for Membrane Trafficking, Department of Neurosciences, Vlaams Instituut Voor Biotechnologie (VIB) Center for Brain and Disease Research, KU Leuven, Leuven, Belgium; 6 Present Address: Department of Chemistry, Molecular Sciences Research Hub, Imperial College London, London, UK; 7 Ludwig Institute for Cancer Research, Brussels, Belgium; 8 SIGN Unit, de Duve Institute, UCLouvain, Brussels, Belgium; 9 Genetic and Epigenetic Alterations of Genomes Unit, de Duve Institute, UCLouvain, Brussels, Belgium; 10 Present Address: CENTOGENE GmbH, 18055 Rostock, Germany; 11 Laboratory for Cognitive Neurology, Department of Neurosciences, Leuven Brain Institute (LBI), KU Leuven (University of Leuven), Leuven, Belgium; 12 Department of Neurology, University Hospital Leuven, Leuven, Belgium; 13 Department of Neurology, University of Ulm, Ulm, Germany; 14 Department of Geriatrics, University Medical Center Göttingen, Göttingen, Germany; 15 Walloon Excellence in Life Sciences and Biotechnology (WELBIO), Brussels, Belgium; 16 Nuffield Department of Medicine, Ludwig Institute for Cancer Research, Oxford University, Oxford, UK; 17 Department of Pathology, University Hospital Leuven, Leuven, Belgium

**Keywords:** Cellular senescence, Telomere shortening, Alzheimer’s disease, Intraneuronal Aβ, Autophagy

## Abstract

Aging is the main risk factor for Alzheimer’s disease (AD) and other neurodegenerative pathologies, but the molecular and cellular changes underlying pathological aging of the nervous system are poorly understood. AD pathology seems to correlate with the appearance of cells that become senescent due to the progressive accumulation of cellular insults causing DNA damage. Senescence has also been shown to reduce the autophagic flux, a mechanism involved in clearing damaged proteins from the cell, and such impairment has been linked to AD pathogenesis. In this study, we investigated the role of cellular senescence on AD pathology by crossing a mouse model of AD-like amyloid-β (Aβ) pathology (5xFAD) with a mouse model of senescence that is genetically deficient for the RNA component of the telomerase (Terc^−/−^). We studied changes in amyloid pathology, neurodegeneration, and the autophagy process in brain tissue samples and primary cultures derived from these mice by complementary biochemical and immunostaining approaches. Postmortem human brain samples were also processed to evaluate autophagy defects in AD patients. Our results show that accelerated senescence produces an early accumulation of intraneuronal Aβ in the subiculum and cortical layer V of 5xFAD mice. This correlates with a reduction in amyloid plaques and Aβ levels in connecting brain regions at a later disease stage. Neuronal loss was specifically observed in brain regions presenting intraneuronal Aβ and was linked to telomere attrition. Our results indicate that senescence affects intraneuronal Aβ accumulation by impairing autophagy function and that early autophagy defects can be found in the brains of AD patients. Together, these findings demonstrate the instrumental role of senescence in intraneuronal Aβ accumulation, which represents a key event in AD pathophysiology, and emphasize the correlation between the initial stages of amyloid pathology and defects in the autophagy flux.

**Supplementary Information:**

The online version contains supplementary material available at 10.1186/s40478-023-01578-x.

## Introduction

Aging is known to be the primary driver of many neurodegenerative diseases, including Alzheimer’s disease (AD) [53]. Thus, the current global increase in life expectancy and population aging is accompanied by a rising incidence of these incurable illnesses, creating huge societal and economic costs worldwide that are expected to worsen over the years [23]. Extensive research is needed to understand the molecular mechanisms underlying the shift from healthy to pathological aging. In this regard, cellular senescence emerges as a key biological process linked to aging that can contribute to or cause the appearance of age-related pathologies like AD [5].

Cellular senescence was originally defined in dividing somatic cells as a permanent cell cycle arrest caused by the appearance of critically shortened telomeres due to the “end replication problem”, i.e., the inability of the ends of linear DNA to be replicated completely during DNA synthesis. As a consequence, telomere attrition gradually appears with increasing number of cell divisions, triggering replicative senescence [2, 48]. It has been demonstrated that a variety of stressors, including oncogenic activation, reactive oxygen species (ROS) and mitochondrial dysfunction, can stimulate the conversion into a state of senescence, mostly through the generation of DNA damage [5]. Senescent cells share distinct detectable features, including the activation of the senescence-associated β-galactosidase (SA-β-gal) [28] and the transcriptional upregulation of cyclin-dependent kinase inhibitors (i.e., p16^Ink4a^, p19^Arf^ and p21^waf1/cip1^) [65, 74]. Senescent cells accumulate with age throughout the body and, although playing a protective role by preventing the appearance of cancer cells, they eventually promote tissue deterioration, producing a low-grade but chronic inflammatory context and acquiring a phenotype that prevents their clearance from the tissue in which they reside [14, 114]. Indeed, the harmful impact of senescent cells in the tissue microenvironment might be ascribed to the acquisition of a specific secretome, called senescence-associated secretory phenotype (SASP), which is characterized by the secretion of pro-inflammatory cytokines, chemokines, extracellular-matrix-degrading enzymes and growth factors that promote a chronic inflammatory state [13, 66]. Additionally, autophagy is an important homeostatic mechanism of lysosome-based degradation that has been linked to cellular senescence. Reduced autophagic activity during aging, which might contribute to the accumulation of damaged proteins and organelles, has been correlated to the onset of age-related diseases [71] and increasing evidence suggests that the age-related decrease in autophagy is particularly linked to neuronal cell senescence in animals and humans [107]. Indeed, the blockage of autophagy during cell senescence has been consistently observed in cultured neurons [56, 80].

In the aging brain, replicative senescence should logically occur in dividing cells such as glial cells or neural stem cells. Indeed, markers of cellular senescence have been found in those cell types in the context of aging and neurodegenerative diseases [5]. There is much more debate on whether non-proliferating terminally differentiated cells such as neurons can acquire a senescent phenotype. Still, neurons accumulate high amounts of DNA damage over time that can trigger a senescence-like phenotype, with increased SA-β-gal activity and elevated expression of proinflammatory interleukins [59], but the impact of cellular senescence in the onset of neurodegenerative diseases is only beginning to be understood.

AD is the most frequent neurodegenerative disease. It is characterized by the gradual appearance of cognitive deficits, neurodegeneration, and the disease-defining pathological features consisting in extracellular senile plaques, containing the amyloid-β (Aβ) peptide, and intracellular neurofibrillary tangles, mainly composed of hyperphosphorylated tau [105]. Aβ, which is produced by sequential cleavage of the amyloid precursor protein (APP) by β-secretases and γ-secretases, is a central and early actor in AD pathogenesis, triggering tau pathology and associated neurodegeneration [45, 46]. Extracellular Aβ deposits like senile plaques might represent a relatively innocuous endpoint of the Aβ aggregation process, while soluble and smaller Aβ aggregates show higher toxicity and a greater correlation with cognitive decline and neurodegeneration [58, 76]. Apart from extracellular deposition, early increases in intracellular Aβ levels have been observed in vulnerable neurons of AD human patients [41, 78] and animal models [22, 87, 110], preceding the appearance of Aβ plaques. Additionally, despite the near-ubiquitous expression of APP in the brain, neurodegeneration in AD animal models and human patients seems to be more restricted to specific brain regions in the hippocampus and the cortex, and the molecular basis for this selective vulnerability remains unknown [98].

Studies have demonstrated the presence of senescent cells in the brains of AD patients and mouse models [5], and the pharmacological and genetic elimination of senescent cells has shown therapeutic benefits for AD pathology in mouse models of tau and amyloid pathology [11, 83, 122]. Mouse models displaying features of cellular senescence and AD pathology can provide useful tools to define the role of senescent cell accumulation in AD. But while the relative short lifespan of mouse models offers a clear benefit for performing research in a time-efficient manner, it also confers a strong disadvantage for studying features of pathological aging that are caused by years of progressive build-up of toxins and DNA damage. Telomerase-deficient mice, lacking de novo synthesis of telomeric DNA repeats and therefore unable to maintain telomere length, are a *bona fide* model of accelerated senescence. In particular, mice carrying a homozygous germline deletion of the telomerase RNA component gene (Terc^−/−^) exhibit critically short telomeres and a premature development of age-related pathologies from the second or third generation [8, 18–20, 51, 70, 72, 101, 103], with recent data showing the increased expression of senescence markers in brain regions of these mice [59]. Additionally, recent reports suggested a causal relationship between shorter telomeres and AD risk [102]. Supporting this idea, genetic variations in genes involved in telomere maintenance have been shown to correlate with AD susceptibility [121].

In our study, we hypothesized that senescence is an upstream event in AD onset. If so, it would have an impact on amyloid pathology, the earliest AD lesion. To understand this link between senescence and AD lesions, we crossed Terc^−/−^ mice with 5xFAD mice, a well-characterized model of amyloid pathology [87]. We found that brain cells (including neurons) of 3rd generation Terc^−/−^ mice exhibit increased senescence traits. Accelerated senescence induces an early accumulation of the Aβ peptide inside neurons from the subiculum and cortical layer V, causing their degeneration, while reducing Aβ plaque load at a later disease stage. Our results further indicate that the triggering effect of senescence on intraneuronal Aβ accumulation is related to an autophagy defect, and that an autophagy alteration is detected in brain regions of AD patients where amyloid pathology occurs.

## Materials and methods

### Animals

Terc knockout mice [8] (Strain #004132, The Jackson Laboratory), carrying a germline deletion for the telomerase RNA subunit Terc, and 5xFAD transgenic mice [87] (Strain #034840-JAX, The Jackson Laboratory), expressing human *APP* and *PSEN1* transgenes with a total of five familial AD (FAD) mutations (Swedish [K670N/M671L], Florida [I716V], and London [V717I] mutations in *APP*, and M146L and L286V mutations in *PSEN1*), were bred. All mice were maintained on a C57BL/6 genetic background. Terc^−/−^ mice were intercrossed to obtain different generations (G) of mice, up to G4. To obtain G3Terc^−/−^ 5xFAD mice, heterozygous Terc^+/−^ mice were first crossed with hemizygous 5xFAD mice to generate double mutant Terc^+/−^ 5xFAD mice. These mice were then crossed with Terc^+/−^ mice to produce first generation G1Terc^−/−^ 5xFAD and G1Terc^−/−^ littermates, and their offspring were subsequently crossbred to obtain the later generations. Genotyping was performed by polymerase chain reaction (PCR) analysis on tail biopsy DNA. Animals were housed on a 12 h light/12 h dark cycle in standard animal care facilities with access to food and water ad libitum. Age- and sex-matched littermates were used for analysis.

### Primary neuronal cultures, lentiviral infection and pharmacological treatments

Primary cultures of neurons were obtained from postnatal day 0 pups as previously described [24, 90], with some modifications. Briefly, brain tissues (containing cortices and hippocampi) were isolated by dissection in ice-cold HBSS/0.2% glucose and meninges were removed. Tissues were then incubated at 37 °C for 3 min in a solution of HBSS/0.2% glucose with 10 mg/ml trypsin (Worthington Biochemical) and 1 mg/ml Deoxyribonuclease I (Worthington Biochemical), and then dissociated in Neurobasal™ medium containing 0.5 mg/ml Deoxyribonuclease I by pipetting up and down with a glass pipette. Dissociation was repeated with a flame-narrowed glass pipette and samples were allowed to sediment for 5 min. Supernatants containing isolated neurons were carefully placed on top of fetal bovine serum (FBS) and centrifuged at 1000× g for 10 min. Pellets were resuspended in Neurobasal™ medium supplemented with 1 mM L-glutamine, 2% B-27™ Supplement and 0.1% penicillin–streptomycin. Cultures were produced individually from each pup. Cells were plated in 24-,12- or 6-well plates pre-coated with 10 μg/ml of poly-L-lysine (Sigma-Aldrich) and maintained at 37 °C in a humidified atmosphere containing 5% CO2.

Lentiviruses were used to study intraneuronal Aβ accumulation in mouse primary neurons. The sequence encoding human APP695 carrying 3 FAD mutations (Swedish [K670N/M671L], Florida [I716V], and London [V717I]) was cloned into the pLVX-CMV bicistronic retroviral vector, to produce the hAPP3xmut plasmid. Lentiviral particles were generated as previously described [24, 100]. Briefly, HEK293T cells were transfected with the hAPP3xmut, pMD2.G (Addgene #12259), and pCMV-dR8.2 (Addgene #12263) plasmids. 48 h after transfection, media and cells were harvested and centrifuged at 1500× g for 10 min at 4 °C. The supernatant was filtered and concentrated with LentiX™ Concentrator reagent following manufacturer’s instructions (Clontech). Primary neurons were infected at DIV 7 and after 24 h, the whole medium was replaced with fresh medium. Non-infected cultures were subjected to the same media change.

To study the autophagic flux, neurons were treated at DIV 14 with either vehicle (water) or chloroquine (25 μM) for 6 h. To evaluate the effects of autophagy activation, neurons transduced with lentiviruses expressing hAPP3xmut were treated at DIV 7 with either vehicle (DMSO) or rapamycin (2.7 nM) for 4 days.

### Postmortem human brain tissue

Brain tissue samples from 39 patients were included in this study (Additional File 1: Table S1). Selection criteria were as previously described [79]. Autopsies were performed in university or municipal hospitals in Belgium (Leuven) and Germany (Bonn, Ulm and Offenbach) in accordance with the applicable laws. Informed consent was obtained following local legislation. The recruitment protocols for the collection of human brains were approved by the ethical committees of UZ Leuven (Belgium; S-59292, S-52971) and the University of Ulm (Germany; 54/08). Neuropathological evaluation of AD pathology in these samples was performed as previously described [79], and based on standardized clinicopathological criteria, including Aβ plaque deposition in the medial temporal lobe (AβMTL phase) determined by Aβ immunohistochemistry [111, 112], the distribution of NFTs in the brain (Braak NFT stage) [9, 10] and neuritic plaque frequency (the Consortium to Establish a Registry for AD (CERAD) score) based on pTau immunohistochemistry [77]. The National Institute of Aging-Alzheimer Association (NIA-AA) score was determined based on the AβMTL phase, Braak-NFT stage and CERAD score, reflecting the degree of AD pathology [55]. The clinical Dementia Rating (CDR) score [54] was retrospectively assessed based upon clinical files as previously described [49], reflecting the stage of cognitive and functional impairment of patients. Cases were classified into three groups based on the clinical and post-mortem neuropathological diagnosis: (1) AD = intermediate-high NIA-AA score, signs of cognitive decline (CDR ≥ 0.5); (2) p-preAD = low–high NIA-AA score, no signs of cognitive decline (CDR = 0); and (3) non-AD = no signs of AD pathology (NIA-AA score = 0), no signs of cognitive decline (CDR = 0). Primary age-related tauopathy (PART) [25] was diagnosed in four non-AD control cases. Relevant clinicopathological characteristics of the cases included in this study are summarized in Additional file 1: Table S1.

Autophagy analysis in these tissue samples was approved by the UZ-Leuven ethical committee (S-64378) and has been carried out in the Laboratory of Neuropathology at KU Leuven.

### Telomere length analysis by quantitative PCR

Cortical brain tissue or primary neurons obtained from mice were digested with proteinase K (100 μg/ml) in lysis buffer (100 mM NaCl, 100 mM Tris–HCl [pH 8.0], 1 mM EDTA [pH 8.0] and 1% SDS) at 50 °C overnight. DNA was purified using the phenol/chloroform method. To measure the length of mouse telomeres, a quantitative PCR method was used as previously described [12], adapted from the method first developed by Cawthon et al. [17]. Two separate quantitative PCR reactions were performed, one with telomere primers and the other with single copy gene primers. Forward and reverse primers (Sigma) targeting the telomere region and the *36B4* gene (single copy gene) are described in Additional file 1: Table S2. The relative telomere length (presented as T/S ratio) was calculated by comparing telomere amplification (T) to that of the single copy gene (S) using the 2^−ΔΔCT^ method [17].

### Quantitative RT-PCR

Total RNA was extracted using TriPure™ Isolation reagent (Roche) according to the manufacturer’s protocol. RNA was resuspended in DEPC-treated water and concentration measured (OD 260 nm) on BioSpec-nano spectrophotometer (Shimadzu Biotech). Reverse transcription (RT) was carried out with the iScript cDNA synthesis kit (Bio-Rad Laboratories) using 1 μg of total RNA in a total volume reaction of 20 μl. Quantitative PCR was performed for the amplification of cDNAs using the appropriate primers (Sigma-Aldrich, see Additional file 1: Table S3) and the GoTaq® qPCR Master Mix (Promega), following manufacturer’s instructions. For negative controls, the iScript reverse transcriptase was omitted in the cDNA synthesis step. Relative quantification was calculated by the 2^−ΔΔCT^ method using *Gapdh* as housekeeping gene. Results were then normalized (fold change) to the control condition.

### Protein isolation

Mouse brain tissue was homogenized by sonication in cold lysis buffer containing 20 mM Tris base (pH 8.0), 150 mM NaCl, 1% NP-40, 10% glycerol and supplemented with protease and phosphatase inhibitor cocktails (Roche). Samples were centrifuged at 16,000 g for 20 min and the supernatants collected and stored at – 80 °C. Additionally, for analysis of insoluble Aβ content, the pellets obtained (containing detergent-insoluble material) were solubilized in 5 M guanidine hydrochloride (GuHCl) by sonication. Samples were centrifuged at 16,000 g for 15 min and the supernatants collected and stored at – 80 °C.

For the analysis of cellular lysates, cells were collected in cold lysis buffer containing 50 mM Tris base (pH 7.5), 150 mM NaCl, 2 mM EDTA, 1% NP-40 and supplemented with protease and phosphatase inhibitor cocktails (Roche). Samples were sonicated and centrifuged at 10,000 g for 10 min and the supernatants collected and kept at – 80 °C.

For the analysis of human brain samples, frozen tissue of the temporal cortex from a subset of cases, AD (n = 5), p-preAD (n = 5) and non-AD (n = 5) cases, was used for protein extraction. Samples were homogenized with a micropestle in extraction buffer (2% SDS in 50 mM Tris, 150 mM NaCl, pH 7.6; TBS) containing nucleases (Thermo Fisher Scientific) and a protease and phosphatase inhibitor cocktail (Thermo Fisher Scientific). Sonication was performed using an ultra-sonic homogenizer (Biologics Inc.), followed by centrifugation with a 5415R centrifuge (Eppendorf) at 13,000× g for 30 min at RT. The supernatant (fraction containing soluble and dispersed proteins) was collected.

### Western blot

Following determination of protein concentration using the Pierce™ BCA Protein assay kit (Invitrogen, Thermo Fisher Scientific), protein extracts (10–30 µg) were mixed with NuPAGE™ LDS Sample Buffer (Invitrogen, Thermo Fisher Scientific) and 50 mM dithiothreitol (DTT), and were heated for 10 min at 70 °C. Samples were resolved by SDS-PAGE electrophoresis on precast NuPAGE™ 4–12% Bis–Tris gels (Thermo Fisher Scientific) and MOPS-SDS or MES-SDS running buffer (Thermo Fisher Scientific), using SeeBlue™ Plus2 pre-stained (Thermo Fisher Scientific) as standard. Samples were then transferred for 2 h at 30 V with NuPAGE™ transfer buffer (Thermo Fisher Scientific) onto 0.1 μm or 0.45 μm nitrocellulose membranes (Thermo Fisher Scientific). After incubation for 30 min in blocking buffer containing 5% non-fat powdered milk in PBS/0.1% Tween®20 (PBS-T), membranes were blotted overnight at 4 °C with primary antibodies (Additional file 1: Table S4) diluted in PBS-T. Horseradish-peroxidase (HRP)-conjugated species-specific secondary antibodies (Sigma-Aldrich) were used to bind primary antibodies prior to ECL detection. ImageJ software (National Institutes of Health) was used to quantify protein band intensity, normalized to the intensity of Actin.

Only for human samples, protein extracts were mixed with NuPAGE™ LDS Sample Buffer (Invitrogen, Thermo Fisher Scientific) and NuPAGE™ Sample Reducing Agent (Invitrogen, Thermo Fisher Scientific), followed by 10 min heating at 75 °C. Samples were loaded onto precast NuPAGE™ 4–12% Bis–Tris Protein Gels (Life Technologies) in equal amounts of protein (20 μg). Electrophoresis was performed with MOPS-SDS or MES-SDS running buffer (Alfa Aesar) at 140 V for 70 min, followed by semi-wet transfer onto Amersham Protran 0.2 µm nitrocellulose membranes (GE Healthcare). Membranes were blocked with 5% non-fat dried milk in PBS-T for 1 h at RT and incubated with primary antibody (Additional file 1: Table S5) in PBS-T overnight at 4 °C. HRP-conjugated species-specific secondary antibodies (Dako) were used to bind primary antibodies. Protein bands were detected using SuperSignal West Pico or Dura chemiluminescent substrate (Thermo Fisher Scientific) and imaged using an Amersham Imager 600 (GE Healthcare). Restore Western Blot Stripping Buffer (Life Technologies) was used to strip bound antibody, followed by incubation with anti-glyceraldehyde-3-phosphate dehydrogenase (GAPDH) primary antibody to confirm equal protein load. ImageJ software was used to quantify protein band intensity, normalized to the intensity of GAPDH.

### Electrochemiluminescence immunoassay (ECLIA) for Aβ quantification

Aβ peptides were measured in protein extracts from primary neurons or brain tissue using the human Aβ 6E10 multiplex ECLIA assay (Meso Scale Discovery) as previously described [43]. For soluble Aβ quantifications from mouse brains, samples were diluted 25 times. For insoluble Aβ quantifications from mouse brains, samples were diluted 1000 times.

### Immunofluorescence stainings

#### Mouse brain slices

Mice were transcardially perfused with PBS and brains were fixed by immersion in PBS/4% paraformaldehyde for 24 h, cryoprotected in PBS/30% sucrose with 0.02% sodium azide, and frozen. Coronal brain sections (30 µm thick) were obtained using a freezing microtome and were placed in cryoprotectant solution (containing 30% ethylene glycol, 20% glycerol) and kept at – 20 °C until further use. Free-floating sections were rinsed three times in PBS and blocked/permeabilized for 30 min at room temperature in PBS containing 3% BSA and 0.5% triton X-100. Brain slices were then incubated overnight at 4 °C with primary antibodies (Additional file 1: Table S4) diluted in PBS containing 3% BSA and 0.5% triton X-100. After three washes with PBS, slices were incubated for 1 h at room temperature with DAPI (1:10,000) and the appropriate fluorophore-conjugated secondary antibodies (Additional file 1: Table S6, dilution 1:500). Finally, slices were washed three times in PBS and mounted in Superfrost™ slides, allowed to dry, and coverslipped with Mowiol. Alternatively, for slices used to evaluate amyloid plaques, incubation with Thioflavin T was additionally performed following the immunofluorescence protocol. After samples were mounted on Superfrost™ slides, they were incubated for 15 min in the dark with 0.01% Thioflavin T (ThT, Sigma-Aldrich) diluted in 50% ethanol. Finally, the sections were washed three times with 80% ethanol, and once with distilled water. Coverslips were then mounted with Mowiol.

For all experiments, images were acquired with an EVOS™ FL Auto fluorescence microscope. For each mouse, four to five coronal brain sections spaced 360 μm apart (approximately in the range from − 1.2 to − 2.8 mm relative to bregma) were processed. For specific analyses of the subiculum region, coronal brain sections were taken between bregma − 2.6 mm and − 3.8 mm. The number of mice per group is indicated in each Figure legend. For intraneuronal Aβ42 evaluation, 3 to 5 images were taken at 20X magnification in the subiculum or cortical regions from each brain slice. The number of cells displaying clear intracellular Aβ42 immunoreactivity per image was manually counted as a measure of neuronal Aβ42 accumulation. For amyloid plaque evaluation, 1 image per slice was taken at 4X magnification. For each picture, the hippocampal region or the cortical region was delineated and ThT and Aβ42 double-positive dots were manually counted as a measure of Aβ42-containing plaques [115]. For glial measurements, 1 image per slice was taken at 4X (for total hippocampus evaluation) or 10X (for other areas) and the percentage of area occupied by Iba-1 or GFAP immunoreactivity was calculated as a readout of glial activation using the ImageJ software, as described elsewhere [1]. Briefly, the investigated area was first traced using ImageJ (for 4X images of the hippocampus). Images were then converted to black-and-white images, and a fixed threshold was established for each experiment to determine the percentage of immunoreactive stained area. For neuronal density evaluation, 3 images/section were taken at 20X (for subiculum and cortex layer V regions) or at 40X (for CA/DG regions) and NeuN-positive nuclei were manually counted in areas delineated using ImageJ.

As negative controls, samples were processed as described in the absence of primary antibody and no signal was detected.

#### Cells

Cells were carefully washed with PBS and fixed with PBS/4% paraformaldehyde for 10 min. Cells were rinsed three times with PBS and then permeabilized with a solution of PBS/0.3% Triton for 30 min and non-specific sites were blocked with PBS/0.3% Triton/5% FBS for 30 min. Primary antibodies (Additional file 1: Table S4) diluted in the blocking solution were incubated overnight at 4 °C. After three washes with PBS, cells were incubated with DAPI (Sigma-Aldrich, dilution 1:10,000) and secondary antibodies (Additional file 1: Table S6, dilution 1:500) diluted in the blocking solution. Finally, samples were consecutively washed in PBS and water, and mounted with Mowiol.

Images were acquired with an EVOS™ FL Auto fluorescence microscope. Aβ42 immunoreactivity was quantified by analysis of integrated optical density with ImageJ software. As negative controls, samples were processed as described in the absence of primary antibody and no signal was detected.

### Immunohistochemistry on human cases

The presence of LC3 and p62 was examined in tissue sections of the medial temporal lobe covering the hippocampus and temporal cortex (Brodmann area BA 36). Samples were embedded in paraffin and microtomed at 5 μm. Tissue was mounted on Flex IHC adhesive microscope slides (Dako) and dried at 55 °C. Tissue sections were processed and stained using a Leica BOND-MAX Automated IHC/ISH Stainer (Leica Biosystems) with the Bond Polymer Refine Detection kit (DS9800, Leica Biosystems) according to the manufacturer’s protocol. Briefly, tissue sections were deparaffinized and chemically treated for epitope retrieval (pH 6.1). Hydrogen peroxide was used to quench the endogenous peroxidase activity. Tissue sections were then incubated with an anti-LC3 and an anti-p62 primary antibody (Additional file 1: Table S5) for 30 min, followed by HRP-labelled secondary antibodies. The substrate chromogen 3,3’-Diaminobenzidine tetrahydrochloride hydrate (DAB) was used to visualize the complex, followed by counterstaining with hematoxylin. After dehydration using an autostainer (Leica Biosystems), sections were mounted using an automated cover-slipper (Leica Biosystems) and Leica CV mount (Leica Biosystems). Images were taken with a Leica DFC7000 T camera (Leica Microsystems) on a Leica DM2000 LED light microscope (Leica Microsystems). Image processing was performed with ImageJ software and Inkscape (https://inkscape.org/).

### Senescence-associated β-galactosidase (SA-β-gal) staining for neurons

Primary cultures of neurons at DIV 14 were used. After removing the media, cells were carefully washed with PBS and fixed for 5 min in PBS/4% paraformaldehyde at room temperature. Cells were washed again in PBS and incubated overnight for 18 h at 37 °C with staining solution prepared in phosphate buffer pH 5.5 and containing 5 mM potassium ferrocyanide, 5 mM potassium ferricyanide, 150 mM NaCl, 2 mM MgCl_2_, and 1 mg/mL X-gal. Cells were then washed and processed for MAP2 immunostaining as described below.

### Statistical analysis

Statistical analyses were carried out using the GraphPad Prism 8.0 software. Normality was assessed with the Shapiro–Wilk test. A parametric test was performed if the data followed a normal distribution. Otherwise, a non-parametric test was used. When two groups were compared, parametric Student’s-t-test or non-parametric Mann–Whitney tests were used. When more than two groups were compared, parametric One-way or Two-way ANOVA with indicated post-hoc tests or non-parametric Kruskal–Wallis were used. Significance is indicated as: ns = non-significant, *P < 0.05, **P < 0.01, ***P < 0.001. The number of independent biological samples is indicated in figure legends with “n = ”.

## Results

### Telomere attrition induces markers of cellular senescence in mouse brain cells

**Fig. 1:**
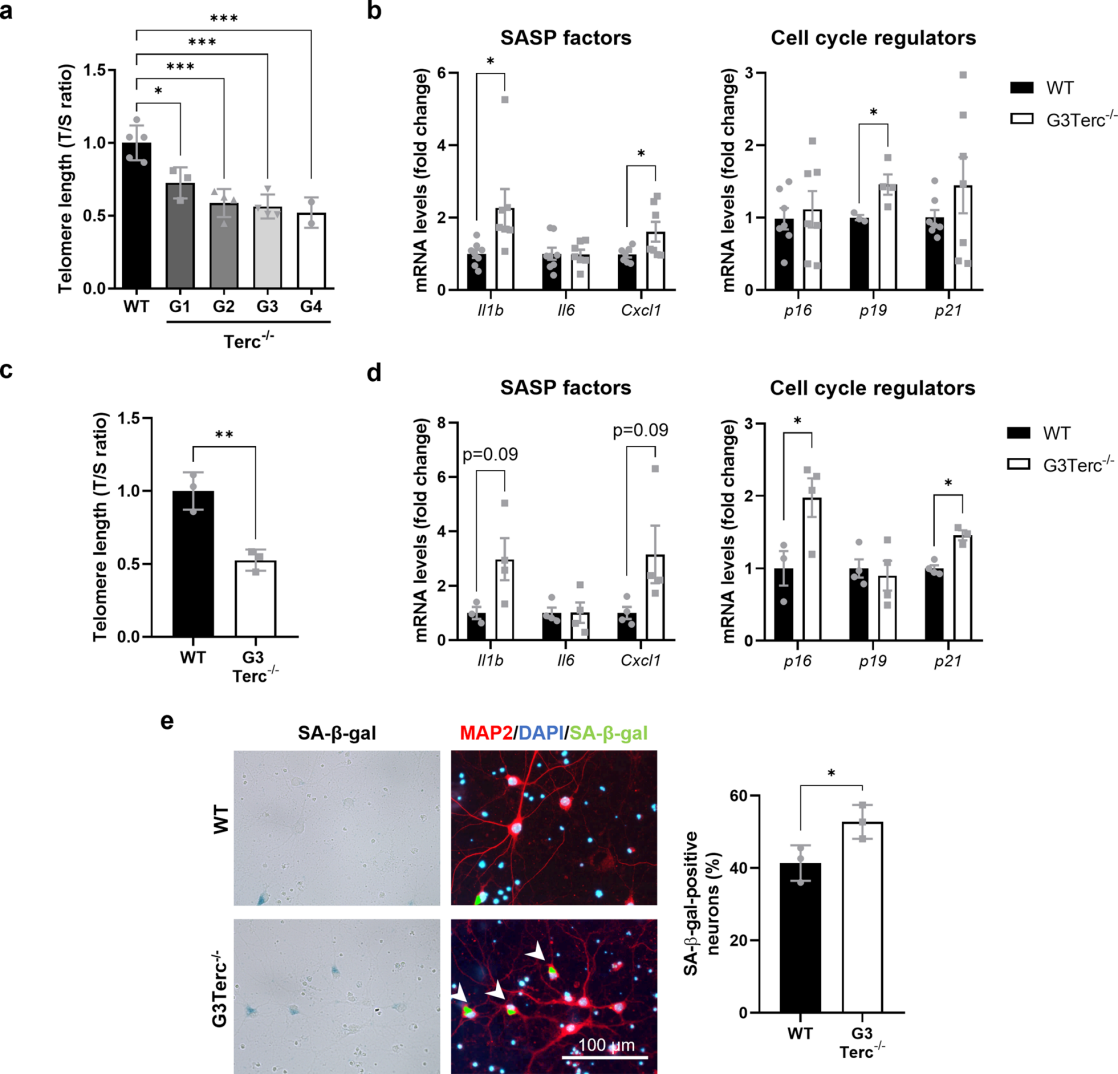
Telomere shortening enhances classical markers of cellular senescence in brain cells. **a** qPCR analysis of telomere length in cortical DNA samples from WT and successive generations (G1-G4) of Terc^−/−^ mice. Average telomere length was calculated as the ratio (T/S) of the telomere repeat copy number (T) to a single copy gene copy number (S = *36B4*). *P < 0.05, ***P < 0.001 (One-way ANOVA with Tukey’s post-hoc analysis, n = 2–5 mice/group). **b** mRNA levels of the SASP factors Il1b, Il6, Cxcl1, and the cyclin-dependent kinase inhibitors p16^Ink4a^ (p16), p19^Arf^ (p19) and p21^waf1/cip1^ (p21), were measured by RT-qPCR in cortical extracts from 5-month-old WT and G3Terc^−/−^ mice. *P < 0.05 (two-tailed Student’s *t-*test, n = 4–8). **c** qPCR analysis of telomere length (T/S ratio) in primary neurons derived from WT and G3Terc^−/−^ mice. **P < 0.01 (two-tailed Student’s *t-*test, n = 3 cultures/group). **d** mRNA levels of Il1b, Il6, Cxcl1, p16^Ink4a^ (p16), p19^Arf^ (p19) and p21^waf1/cip1^ (p21), were measured by RT-qPCR in WT and G3Terc^−/−^ primary neurons. *P < 0.05 (two-tailed Student’s *t-*test, n = 3–4 cultures/group). **e** Primary neurons obtained from WT and G3Terc^−/−^ mice were stained for SA-β-gal, followed by immunostaining using the selective neuronal marker MAP2 (red), and the percentage of SA-β-gal-positive neurons was calculated. *P < 0.05 (two-tailed Student’s *t-*test, n = 3 cultures/group). Scale bar: 100 μm. All data are presented as the mean ± SEM

Telomere attrition was initially described as the main inducer of cellular senescence. To validate our model of telomere-induced senescence, telomere length was first measured using the qPCR method (T/S ratio) in cortical tissue collected from wild-type (WT) mice and from several generations of intercrossings of Terc^−/−^ mice (G1-G4 for generation 1 to generation 4) at 5 months of age. As expected, progressive telomere attrition was observed in successive generations of Terc^−/−^ mice, compared to WT mice, with already significant differences appearing from the first generation (G1) and reaching a plateau at G3-G4 (Fig. 1a).

Telomeres in laboratory mice are significantly longer than human telomeres and therefore require more cell divisions to reach a critical length that can trigger a senescent phenotype [8, 62]. We thus decided to carry on further experiments on the third generation of Terc^−/−^ mice referred to herein as G3Terc^−/−^. We first characterized the presence of a panel of known senescence markers, including the SASP factors Il1b, Il6, Cxcl1, and the cyclin-dependent kinase inhibitors p16^Ink4a^ (p16), p19^Arf^ (p19) and p21^waf1/cip1^ (p21), in 5-month-old WT and G3Terc^−/−^ mice. A significant upregulation in *Il1b*, *Cxcl1* and *p19* mRNA levels, as determined by RT-qPCR, was observed in the cortex of G3Terc^−/−^ mice (Fig. 1b). Evidence of cell senescence was then investigated using primary neurons from WT and G3Terc^−/−^ mice. Neurons bearing shorter telomeres, as evaluated by qPCR (Fig. 1c), exhibit a transcriptional upregulation of *Il1b*, *Cxcl1*, *p16* and *p21* (Fig. 1d). Additionally, G3Terc^−/−^ primary neurons presented higher SA-β-gal staining, which is a widely accepted marker of senescence (Fig. 1e). Non-neuronal cells from telomerase-deficient mice also exhibited features of senescence, as evidenced by a striking increase in the percentage of SA-β-gal-positive astrocytes (Additional file 1: Fig. S1). Overall, our results indicate that cellular senescence readily occurs in the brains of G3Terc^−/−^ mice when compared to WT mice, both in neuronal and in non-neuronal cells.

### Accelerated senescence enhances early intraneuronal Aβ accumulation

The accumulation and oligomerization of the Aβ peptide is an early event in AD pathology [45, 46]. Additionally, it has been shown that intraneuronal Aβ accumulation in specific AD-vulnerable neurons precedes the formation of extracellular Aβ plaques both in AD patients [41, 78] and mouse models [22, 87, 110]. To investigate the impact of telomere-induced senescence on amyloid pathology found in AD, we crossed the Terc^−/−^ mice with the well-described 5xFAD transgenic AD mouse model that expresses human *APP* and *PSEN1* transgenes with a total of five FAD mutations: the Swedish (K670N/M671L), Florida (I716V), and London (V717I) mutations in *APP*, and the M146L and L286V mutations in *PSEN1*. Four different genotypes were generated to be used in our study: WT mice used as controls, G3Terc^−/−^ mice, 5xFAD mice, and the novel G3Terc^−/−^ 5xFAD mice.

**Fig. 2:**
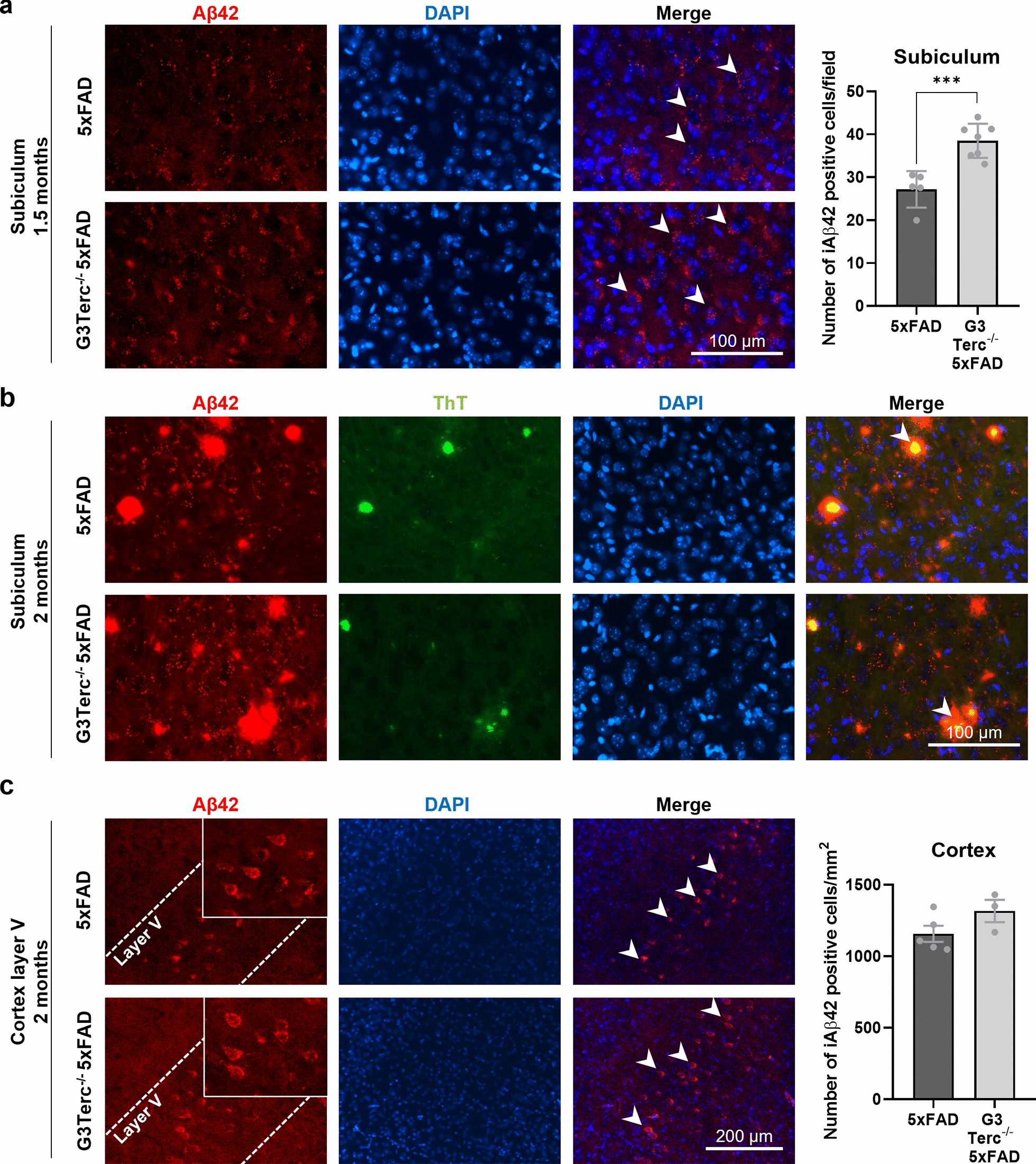
Accelerated senescence enhances aberrant intracellular Aβ accumulation in 5xFAD mice. **a** Immunostaining analysis of Aβ42 (Aβ42 antibody, clone H31L21, red) in the subiculum from 1.5-month-old 5xFAD and G3Terc^−/−^ 5xFAD mice. Representative photomicrographs are shown. Quantitative analysis of intracellular Aβ42 was performed by counting cells displaying intracellular Aβ42 staining (arrows). Scale bar: 100 μm. ***P < 0.001 (two-tailed Student’s *t-*test, n = 5–7 mice/group). **b** Immunostaining analysis of Aβ42 and Aβ42-containing plaques (Aβ42 antibody, clone H31L21, red; Thioflavin T dye, green, colocalization indicated by arrows) in the subiculum from 2-month-old 5xFAD and G3Terc^−/−^ 5xFAD mice. Representative photomicrographs are shown. Scale bar: 100 μm. **c** Immunostaining analysis of Aβ42 (Aβ42 antibody, clone H31L21, red) in the cortical layer V region from 2-month-old 5xFAD and G3Terc^−/−^ 5xFAD mice. Representative photomicrographs are shown. Quantitative analysis of intracellular Aβ42 was performed by counting cells displaying intracellular Aβ42 staining (arrows). Scale bar: 200 μm. Non-significant (two-tailed Student’s *t-*test, n = 3–5 mice/group). All data are presented as the mean ± SEM

To investigate the state of early Aβ pathology in our mouse models, immunohistological analyses were first performed on brain sections from all four groups of mice at 1.5 months of age using an anti-Aβ42 antibody. It has been reported that 5XFAD mice present high levels of the Aβ42 peptide, which starts to accumulate intracellularly at 1.5 months of age, prior to amyloid deposition and gliosis, which begins at two months of age [87]. While WT and G3Terc^−/−^ mice showed no positive signal for Aβ, 5XFAD mice and G3Terc^−/−^ 5xFAD mice exhibited intracellular Aβ staining in cells located within the subiculum. No apparent Aβ plaque staining was observed in any brain region at this early stage. When comparing both genotypes, we found that G3Terc^−/−^ 5xFAD mice showed a significant increase in the number of cells with intracellular Aβ accumulation (Fig. 2a). Apart from the subiculum, early intraneuronal Aβ accumulation in 5xFAD mice was also reported in large pyramidal neurons of cortical layer V [30, 87]. However, given that we did not observe reliable Aβ staining in the cortical layer V of 1.5-month-old 5xFAD and G3Terc^−/−^ 5xFAD mice (Additional file 1: Fig. S2), we performed similar immunostainings analyses at a slightly later time point. Our results show that 2-month-old 5xFAD and G3Terc^−/−^ 5xFAD mice already present many ThT-positive amyloid plaques containing Aβ42 in the subiculum region (Fig. 2b), while a distinct intracellular Aβ staining pattern was observed in the cortical layer V (Fig. 2c). Further quantification suggests a trend towards an increase in the number of cells accumulating Aβ in the cortical layer V in G3Terc^−/−^ 5xFAD mice. To evaluate the type of cells accumulating intracellular Aβ, we assessed the colocalization of Aβ42 immunoreactivity with the neuronal markers NeuN or β-III-Tubulin in 2-month-old 5xFAD mice. All cells in the subiculum and cortical layer V that presented intracellular accumulation of Aβ42 were identified as neurons (Additional file 1: Fig. S3). Taken together, we can conclude that accelerated senescence related to telomere attrition leads to intracellular Aβ42 accumulation in specific brain regions (i.e., subiculum and cortical layer V) prior to extracellular amyloid deposition.

The correct immunolabeling of intraneuronal Aβ is still a matter of debate as it could be associated with technical biases. It has been proven that antibodies raised against N-terminal Aβ residues recognize not only Aβ but also its precursor (APP) and CTFβ, a C-terminal stub produced after the cleavage of APP at the β-site. Both APP and CTFβ are much more abundant than Aβ inside the cells [36], especially in FAD mutant mice (such as 5xFAD mice) expressing the Swedish mutation, which is known to trigger the β-cleavage of APP [31]. Antibodies raised against the C-terminus of Aβ40 and Aβ42 should avoid these cross-reactions and specifically detect Aβ peptides. In order to validate the specificity of the anti-Aβ42 antibody used in our study, Western blot (WB) analyses were performed on hippocampal protein extracts derived from WT and 5xFAD mice at 5 months of age. While the W0-2 antibody (raised against N-terminal anti-human Aβ) recognizes APP, CTFβ and Aβ peptides of human origin in 5xFAD mice, the C-terminal Aβ42 antibody (clone H31L21) only recognizes the Aβ42 peptide in the same samples (Additional file 1: Fig. S4a). Using the same antibodies, immunohistological analyses were performed on brain sections from 5xFAD mice at 5 months of age. In particular, the CA1 hippocampal pyramidal cell layer was investigated as it has been reported to lack intraneuronal Aβ immunoreactivity while presenting elevated levels of intracellular human APP [52, 57]. Our data demonstrated that the W0-2 antibody strongly labels the soma of cells located within the CA1 region, probably due to APP recognition, while specific staining against Aβ42 does not result in any detectable signal (Additional file 1: Fig. S4b). Thus, our results confirmed that positive signals observed in Fig. 2 are not due to antibody cross-recognition patterns but are indeed readily related to Aβ42 accumulation.

**Fig. 3:**
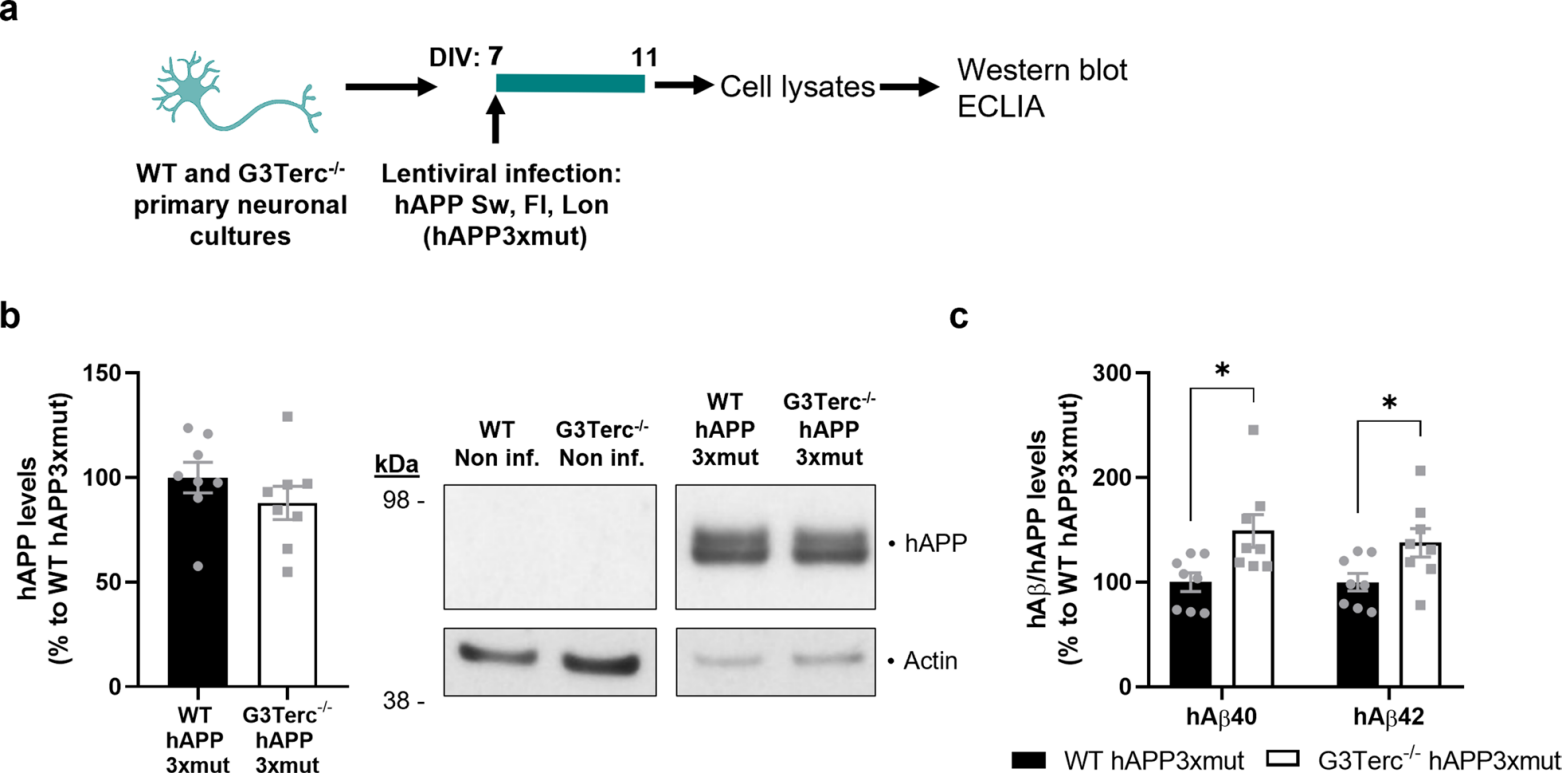
Aberrant intracellular Aβ accumulation in senescent primary neurons overexpressing mutant APP. **a** Schematic illustration of the experimental plan. Primary neuronal cultures were obtained from WT and G3Terc^−/−^ mice and infected at 7 days in vitro (DIV) with lentivirus expressing hAPP3xmut: mutated human *APP* (hAPP) carrying 3 AD-linked mutations (the Swedish [K670N/M671L], Florida [I716V], and London [V717I] mutations). Cell lysates were collected at 11 DIV. **b** Western blot analysis showing hAPP relative protein levels in WT and G3Terc^−/−^ neurons overexpressing APP3xmut. Actin was used as loading control, and the levels in the control group were set as 100%. Non-significant (two-tailed Student’s *t-*test, n = 8 cultures/group). The lack of hAPP detection in non-infected WT and G3Terc^−/−^ neurons is shown. **c** MSD Electro-Chemiluminescence Immuno-Assay (ECLIA) showing relative protein levels of human Aβ40 and Aβ42 in WT and G3Terc^−/−^ neurons overexpressing hAPP3xmut. Results were normalized by the amount of hAPP protein levels obtained in previous Western blot analyses, and the levels in the control group set as 100%. *P < 0.05 (two-tailed Student’s *t-*test or Mann–Whitney test, n = 8 cultures/group). hAβ signal was not detected in non-infected WT and G3Terc^−/−^ neurons. All data are presented as mean ± SEM

The occurrence of intraneuronal Aβ42 accumulation in the telomerase-deficient context was further investigated using an in vitro approach. Human APP expression in primary neurons obtained from 5xFAD mice was weak, and even though human Aβ species were present at detectable levels in the media, intraneuronal Aβ levels were below the detection limit (data not shown). For this reason, primary neuronal cultures from WT and G3Terc^−/−^ pups were transduced with lentiviruses encoding mutated human APP695 bearing the three FAD mutations present in 5xFAD mice: K670N/M671L, I716V, V717I (Fig. 3a). Lentiviral-mediated expression of human APP was confirmed by WB. Densitometric quantification showed no differences in human APP levels between WT and G3Terc^−/−^ primary neurons (Fig. 3b). Since the WB technique is not sensitive enough to detect Aβ in neuronal lysates [61], we quantified intracellular Aβ40 and Aβ42 levels by multiplex electrochemiluminescence immunoassay (ECLIA). Similar to what happens in vivo, our results indicate an intracellular accumulation of both Aβ40 and Aβ42 in G3Terc^−/−^ primary neurons (Fig. 3c).

### Accelerated senescence reduces late Aβ plaque load without altering Aβ generation or Aβ degradation by glial cells

**Fig. 4:**
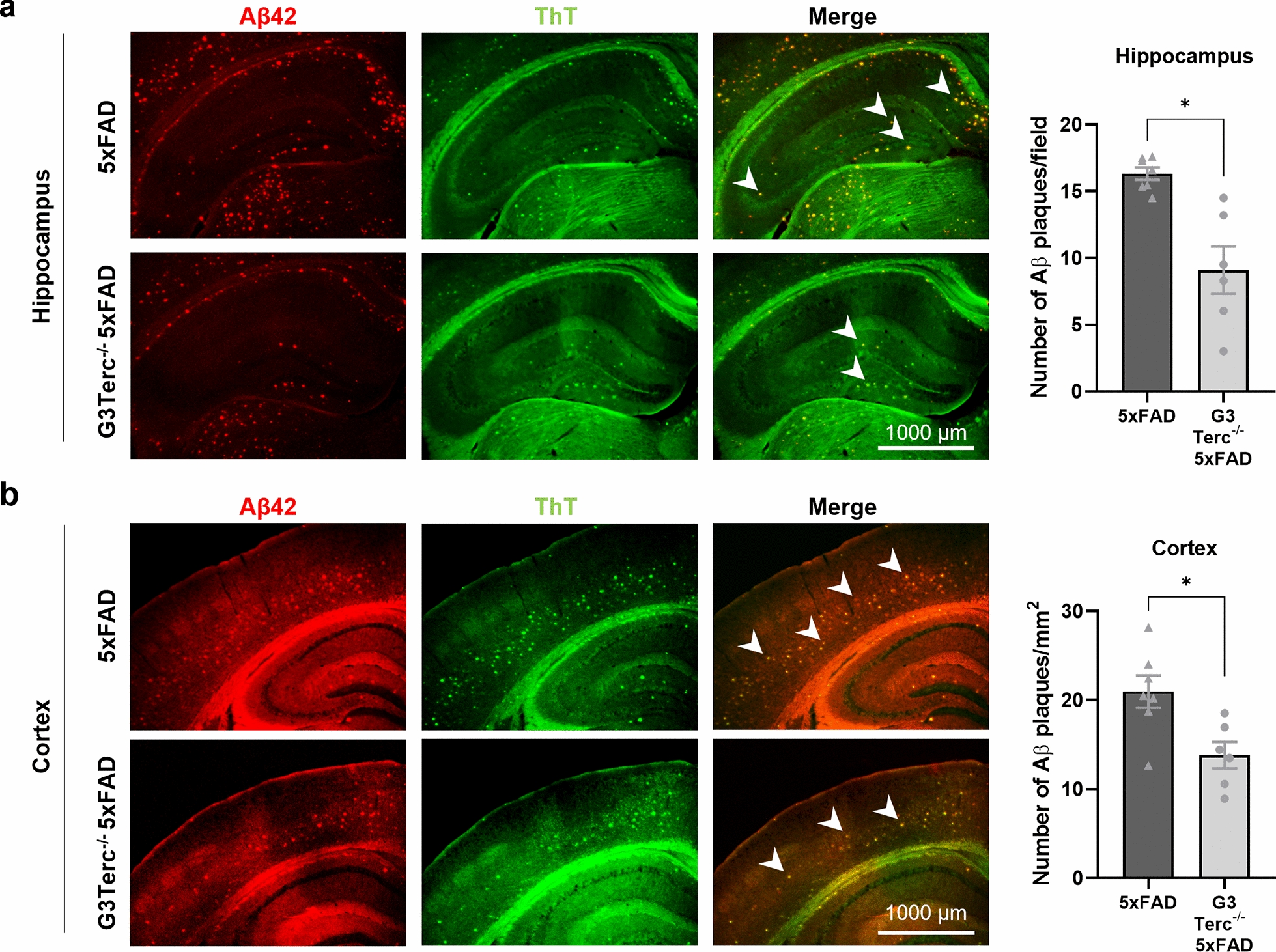
Accelerated senescence reduces Aβ plaque load in 5-month-old 5xFAD mice. Immunostaining analysis of Aβ42-containing plaques (Aβ42 antibody, clone H31L21, red; Thioflavin T dye, green) in the hippocampus (**a**) and cortical (**b**) regions from 5-month-old 5xFAD and G3Terc^−/−^ 5xFAD mice. Representative photomicrographs are shown. Quantitative analysis of Aβ deposition was performed by counting double-positive dots (arrows). Scale bar: 1000 μm. *P < 0.05 (two-tailed Student’s *t-*test, n = 6–7 mice/group). All data are presented as the mean ± SEM

**Fig. 5:**
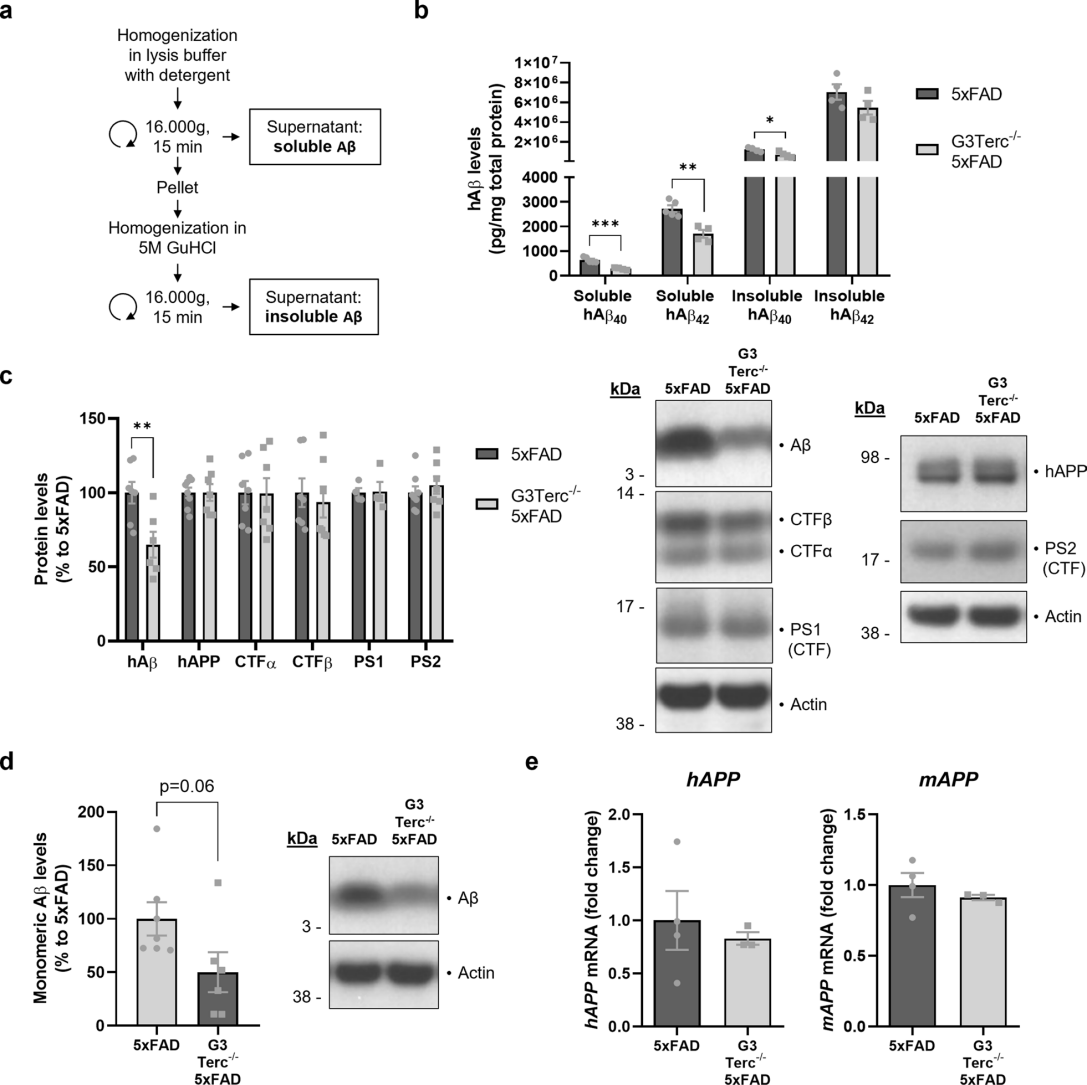
Accelerated senescence reduces Aβ levels in 5-month-old 5xFAD mice without altering APP processing or expression. **a** Schematic diagram depicting the sequential extraction of fractions containing detergent-soluble proteins (containing soluble Aβ) and guanidine hydrochloride (GuHCl)-soluble proteins (containing insoluble Aβ). **b** MSD Electro-Chemiluminescence Immuno-Assay of human Aβ (hAβ) showing soluble and insoluble hAβ40 and hAβ42 levels for mg of total protein in hippocampal extracts from 5-month-old 5xFAD and G3Terc^−/−^ 5xFAD mice. *P < 0.05, **P < 0.01, ***P < 0.001 (two-tailed Student’s *t-*test, n = 4–5 mice/group). **c** Western blot analysis showing relative protein levels of hAβ, hAPP, CTFα, CTFβ, PS1 and PS2 in hippocampal protein extracts from 5-month-old 5xFAD and G3Terc^−/−^ 5xFAD mice. Actin was used as loading control, and the levels in the control group were set as 100%. **P < 0.01 (two-tailed Student’s *t-*test, n = 4–8 mice/group). **d** Western blot analysis showing relative protein levels of hAβ in cortical protein extracts from 5-month-old 5xFAD and G3Terc^−/−^ 5xFAD mice. Actin was used as loading control, and the levels in the control group were set as 100%. *P* = 0.06 (two-tailed Student’s *t-*test, n = 6–7 mice/group). **e** RT-qPCR analyses of mouse *APP* (*mAPP*) and human *APP* (*hAPP*) mRNA levels in total brain extracts from 5-month-old 5xFAD and G3Terc^−/−^ 5xFAD mice. Non-significant (two-tailed Student’s *t-*test, n = 3–4 mice/group). All data are presented as mean ± SEM

Next, we decided to explore the role of accelerated senescence on amyloid pathology at later disease stages, and in particular its impact on the formation of amyloid plaques. Aβ deposition starts to appear in the subiculum of 5xFAD mice from 2 months of age (Fig. 2b), but it can only be observed in the hippocampus and cortical regions of 5xFAD mice from 4 months of age [87]. We collected brain samples from all four genotypes of mice at 5 months of age. Brain sections were processed for histological analysis of amyloid plaque formation by colocalization of the anti-Aβ42 antibody and the Thioflavin T (ThT) dye, which binds to amyloid fibrils. As expected, WT and G3Terc^−/−^ mice showed no positive staining, but 5XFAD mice exhibited substantial plaque burden. We observed a significant reduction of Aβ plaque formation in the hippocampus (Fig. 4a) and cortex (Fig. 4b) of G3Terc^−/−^ 5xFAD mice, compared to 5xFAD mice. At this stage (5 months of age), amyloid pathology was mainly represented by extracellular deposition of insoluble Aβ and not by intracellular accumulation as observed earlier (1.5–2 months of age). To determine the levels of Aβ in its soluble and insoluble forms at 5 months, we performed a sequential extraction (Fig. 5a) on hippocampal homogenates, obtaining two separate fractions containing detergent-soluble proteins (soluble Aβ) or guanidine hydrochloride (GuHCl)-soluble proteins (insoluble Aβ). The levels of two main isoforms of human Aβ (hAβ40 and hAβ42), measured by ECLIA, were found decreased in both soluble and insoluble fractions of G3Terc^−/−^ 5xFAD mice when compared to 5xFAD mice (Fig. 5b). This is consistent with the observed decrease in amyloid load (Fig. 4) and indicates that levels of insoluble aggregated Aβ found in plaques are correlated to the amount of soluble Aβ present in the same brain region. To determine whether the reduction in Aβ load could be primarily due to altered APP expression or processing, WB analyses were performed on soluble protein extracts. Again, Aβ levels were found decreased both in hippocampal (Fig. 5c) and cortical (Fig. 5d) extracts of G3Terc^−/−^ 5xFAD mice, while levels of full-length human APP were not altered (hippocampal extracts, Fig. 5c). In line with this observation, mRNA levels of the human *APP* transgene and the endogenous murine *APP* gene were unchanged between 5xFAD and G3Terc^−/−^ 5xFAD brains (Fig. 5e). Protein levels of APP C-terminal fragments CTFα and CTFβ and presenilins 1 and 2 (PS1 and PS2) were also similar between genotypes (Fig. 4c). Altogether, our results suggest that the senescent context reduces Aβ levels and amyloid load in 5-month-old G3Terc^−/−^ 5xFAD mice without affecting APP expression or processing and therefore Aβ generation.

A potential increase in Aβ degradation by glial cells in the context of telomere attrition could be another mechanism leading to a decrease in Aβ load as previously reported [95]. Indeed, microglia and astrocytes have been shown to play a key role in Aβ clearance and degradation [92]. To explore this possibility, we analyzed the state of the two main glial populations in all groups of mice. Activation of astrocytes and microglia was evaluated by GFAP and Iba-1 immunoreactivity, respectively. WB analysis indicated increased GFAP protein levels in hippocampal and cortical regions of 5xFAD mice (Additional file 1: Fig. S5a), which was further confirmed by immunohistological analyses of the area occupied by GFAP-positive astrocytes (Additional file 1: Fig. S5b). However, telomere attrition and subsequent cellular senescence do not modify astrocyte activation. Similar experiments were carried out to monitor Iba-1 expression by WB (Additional file 1: Fig. S6a) and by immunohistological analyses (Additional file 1: Fig. S6b). Although a trend towards an increase in Iba-1 protein levels was found in G3Terc^−/−^ mice, significant activation of microglia was observed in 5xFAD mice and appeared to the same extent in G3Terc^−/−^ 5xFAD mice. Together, our results indicate that gliosis is primed by amyloid pathology but not aggravated by Terc^−/−^-related senescence, and support the lack of glial contribution on the amyloid load decrease observed in G3Terc^−/−^ 5xFAD mice when compared to 5xFAD mice at the same age (Fig. 4). These results imply that the modification in amyloid deposition observed in our models could be primarily driven by neuronal mechanisms (e.g., alteration of neuronal Aβ accumulation) and not by Aβ clearance associated to non-neuronal cells.

### Intraneuronal Aβ accumulation due to cell senescence correlates with neuronal loss

**Fig. 6:**
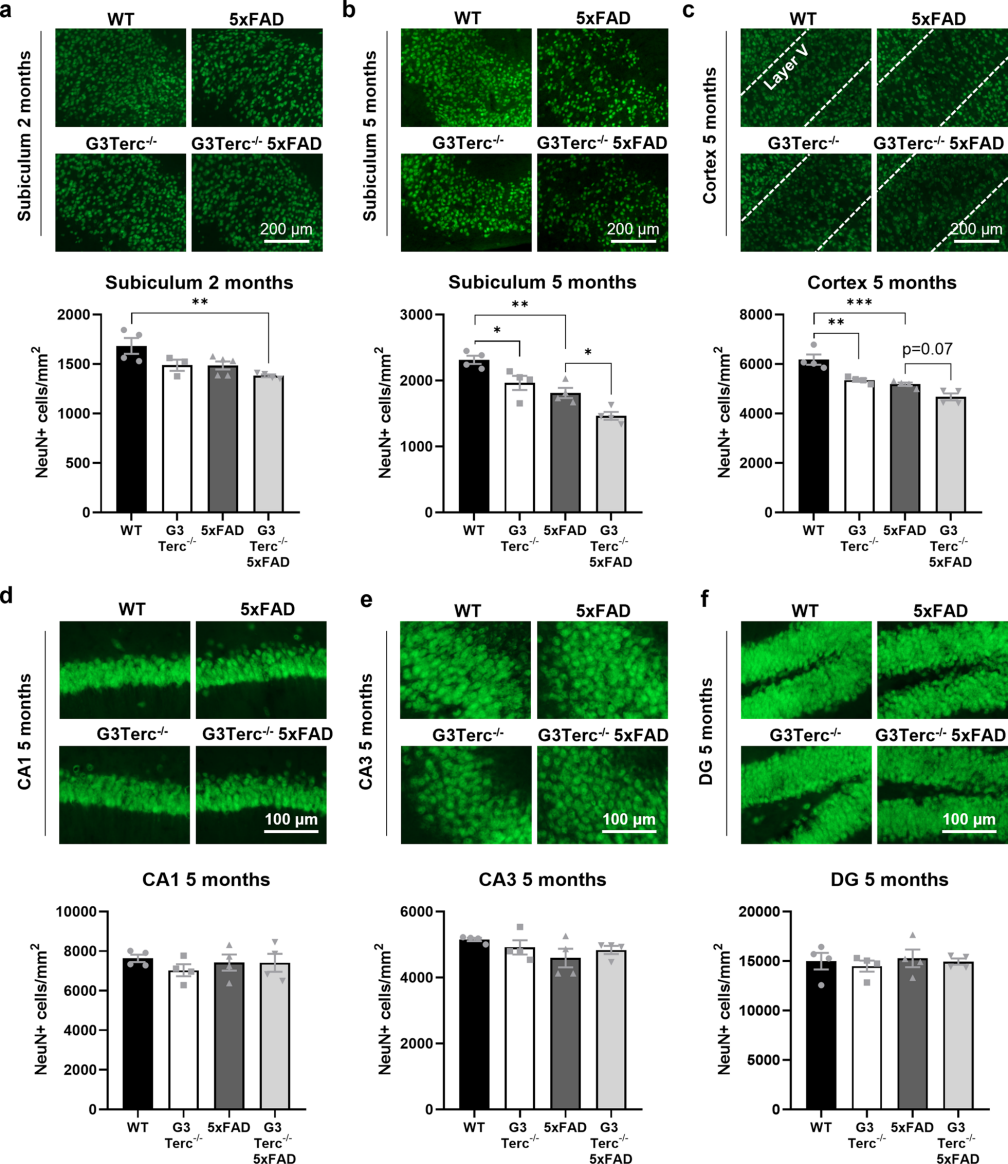
Accelerated senescence in 5xFAD mice decreases neuronal density in brain regions presenting intraneuronal Aβ accumulation. **a** NeuN immunostainings in the subiculum of 2-month-old WT, G3Terc^−/−^, 5xFAD and G3Terc^−/−^ 5xFAD mice. Representative photomicrographs are shown. **P < 0.01 (One-way ANOVA with Tukey’s post-hoc analysis, n = 3–5). NeuN immunostainings in the subiculum (**b**) and cortex (**c**) of 5-month-old WT, G3Terc^−/−^, 5xFAD and G3Terc^−/−^ 5xFAD mice. Representative photomicrographs are shown. Layer V of the cortex is indicated by dashed lines. Scale bar: 200 μm. *P < 0.05, **P < 0.01, ***P < 0.001 (One-way ANOVA with Tukey’s post-hoc analysis, n = 4 mice/group). NeuN immunostainings in the CA1 (**d**) CA3 (**e**) and DG (**f**) region of 5-month-old WT, G3Terc^−/−^, 5xFAD and G3Terc^−/−^ 5xFAD mice. Representative photomicrographs are shown. Scale bar: 100 μm. Non-significant (One-way ANOVA with Tukey’s post-hoc analysis, n = 4 mice/group). The number of NeuN-positive cells per mm^2^ was calculated as a measure of neuronal density. All data are presented as mean ± SEM

Intraneuronal Aβ accumulation was reported to be neurotoxic and induce neuronal apoptosis [30, 61]. In order to evaluate the pathological impact of the intracellular Aβ42 accumulation observed in G3Terc^−/−^ mice, neuronal density was analyzed by immunofluorescent staining using an anti-NeuN antibody. The number of NeuN-positive cells was initially measured in the subiculum of all groups of mice at 2 months of age. Compared to WT mice, a trend towards decreased neuronal density was found in G3Terc^−/−^ and 5xFAD mice, with significant neuronal loss observed in G3Terc^−/−^ 5xFAD mice, suggesting an additive effect for both genotypes (Fig. 6a). Loss of neuronal density was even more apparent when measured in the subiculum of mice aged 5 months, and our results indicate that both telomere attrition and amyloid pathology significantly reduced neuronal density in this area (Fig. 6b). Similarly, the number of NeuN-positive cells in the cortical layer V of 5-month-old mice was significantly reduced in the context of amyloid pathology and accelerated senescence (Fig. 6c). In contrast, hippocampal regions lacking initial intraneuronal Aβ accumulation, such as the CA1, CA3 and dentate gyrus, showed no significant differences in neuron number between groups when evaluated at 5 months of age (Fig. 6d–f), an age at which amyloid plaques can be observed throughout the hippocampus of 5xFAD mice. Our data indicate that telomere-induced senescence triggers neurodegeneration per se in specific brain regions, but that this is aggravated in regions where early intraneuronal Aβ accumulation occurs. Moreover, our results further support the hypothesis that intracellular Aβ, rather than Aβ plaques, are the Aβ species closely associated with neurotoxicity in AD pathology [30, 39, 61].

### Altered autophagy flux caused by cell senescence might underlie abnormal Aβ accumulation and can be detected in postmortem AD brains

Since our findings suggest that Aβ accumulates inside a particular subset of neurons upon telomere-induced senescence, which correlates to reduced extracellular Aβ deposition at a later disease stage, we decided to investigate the potential underlying mechanism of this phenomenon. Evidence indicates that Aβ peptides can accumulate intracellularly while their extracellular release decreases when autophagy is impaired [85, 93]. Additionally, some reports have demonstrated impaired autophagy in cells with critically short telomeres obtained from the 4th generation of Tert and Terc deficient mice [20], and an autophagic flux reduction has been observed in senescent neurons both in vitro and in vivo [80].

**Fig. 7:**
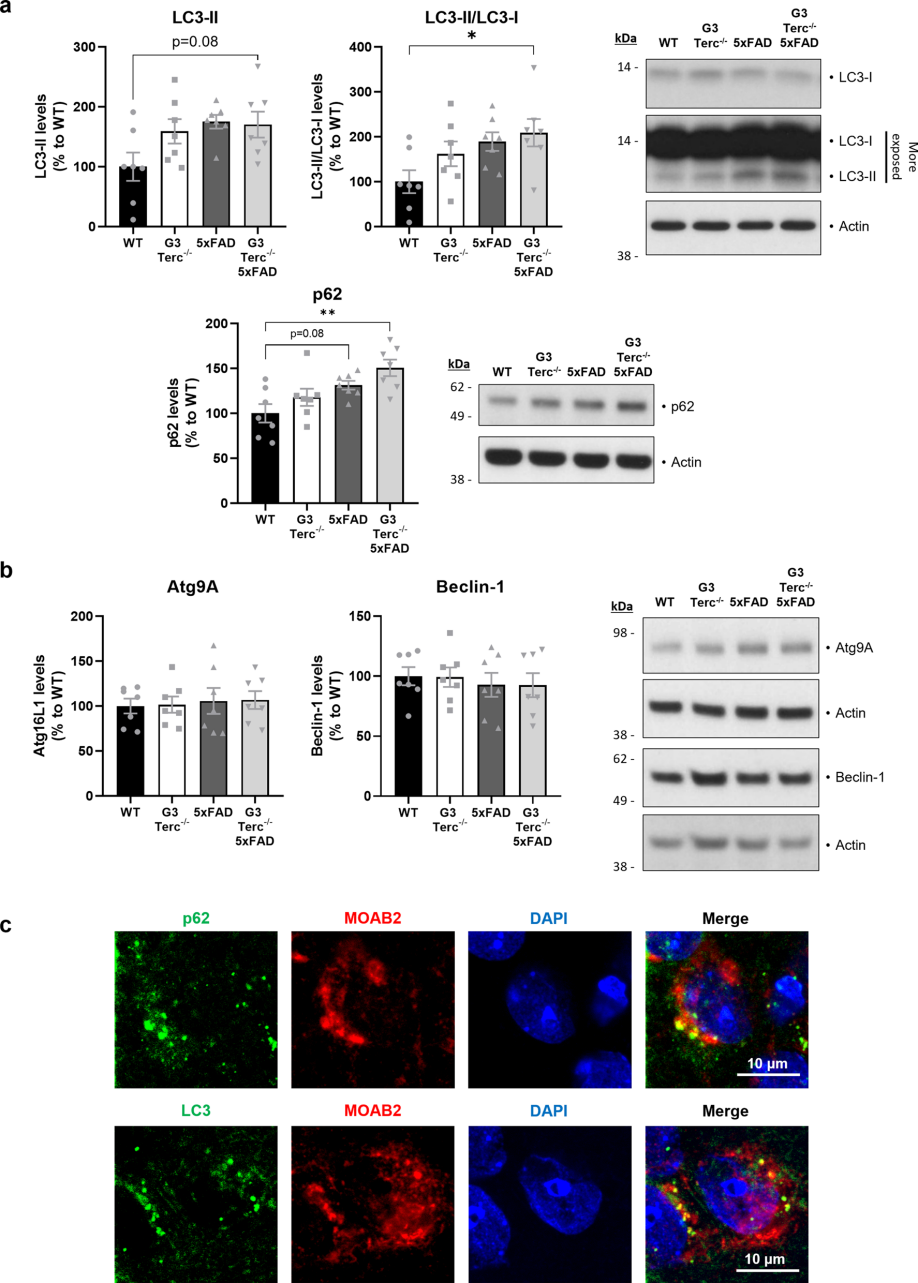
Accelerated senescence alters autophagy in vivo. **a** Western blot analysis showing p62 and LC3 relative protein levels in hippocampal protein extracts from 5-month-old WT, G3Terc^−/−^, 5xFAD and G3Terc^−/−^ 5xFAD mice. Actin was used as loading control, and the levels in the control group were set as 100%. *P < 0.05, **P < 0.01 (One-way ANOVA with Tukey’s post-hoc analysis, n = 7 mice/group). All data are presented as mean ± SEM. **b** Western blot analysis showing Atg9A and Beclin-1 relative protein levels in hippocampal protein extracts from 5-month-old WT, G3Terc^−/−^, 5xFAD and G3Terc^−/−^ 5xFAD mice. Protein levels were normalized by the amount of Actin, and the levels in the control group were set as 100%. Non-significant (One-way ANOVA with Tukey’s post-hoc analysis, n = 7 mice/group). **c** Colocalization of Aβ (MOAB2, red) with two different autophagy markers (p62 or LC3, green) in the subiculum of 2-months-old 5xFAD mice. Representative photomicrographs are shown. Scale bar: 10 μm

**Fig. 8:**
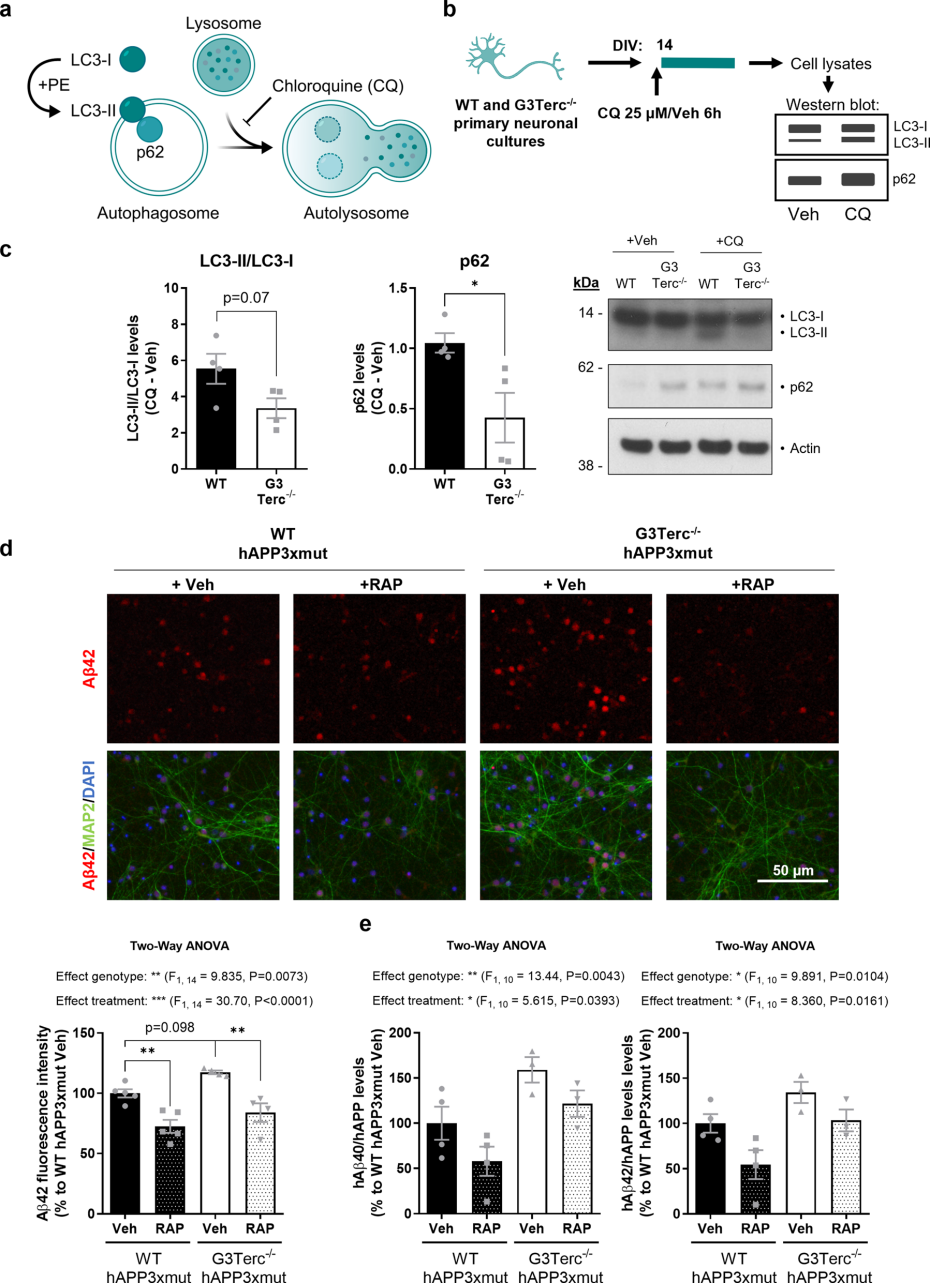
Altered autophagy in senescent primary neurons modulates intraneuronal Aβ accumulation. **a** Illustration depicting the process of autophagy. Chloroquine (CQ) blocks the fusion between the lysosome and the autophagosome. **b** Schematic for the experimental design followed to measure the autophagic flux in vitro. Primary neuronal cultures were obtained from WT and G3Terc^−/−^ mice and treated with CQ (25 μM) or vehicle (Veh) at 14 days in vitro (DIV). Cell lysates were collected after 6 h of treatment for subsequent Western blot analysis. **c** Western blot analysis showing the difference in LC3-II/LC3-I ratio and in p62 relative protein levels after CQ treatment in the cell lysates mentioned in “b”. Actin was used as loading control. Results are expressed as difference between CQ and Veh conditions. *P < 0.05 (two-tailed Student’s *t-*test, n = 4 cultures/group). **d** Primary neurons obtained from WT and G3Terc^−/−^ mice were infected with hAPP3xmut-expressing lentiviruses and treated with rapamycin (RAP) or vehicle (Veh) for 4 days, and intraneuronal Aβ42 accumulation was evaluated by immunostaining analysis of Aβ42 (red) and the neuronal marker MAP2 (green). Relative fluorescence intensity was calculated. **P < 0.01 (Two-way ANOVA with Tukey’s post-hoc analysis, n = 4–5 cultures/group). Scale bar: 50 μm. **e** MSD Electro-Chemiluminescence Immuno-Assay showing human Aβ40 and Aβ42 levels in WT and G3Terc^−/−^ neurons overexpressing hAPP3xmut and treated with rapamycin (RAP) or vehicle (Veh) for 4 days. Results were normalized by the amount of hAPP protein levels obtained in previous Western blot analyses, and the levels in the control group were set as 100%. *P < 0.05, **P < 0.01 (Two-way ANOVA with Tukey’s post-hoc analysis, n = 3–4 cultures/group). hAβ signal was not detected in non-infected WT and G3Terc^−/−^ neurons. All data are presented as the mean ± SEM

Based on this knowledge, we evaluated whether autophagic flux was altered in our mice. This can be achieved by measuring the levels of proteins associated with autophagosomes, such as the microtubule‐associated protein 1A/1B‐light chain 3 (LC3), which is converted from LC3-I to LC3-II during autophagosome formation, and p62 (also called sequestosome 1 or SQSTM1), an autophagy cargo that binds to LC3 [119]. Indeed, accumulation of LC3-II and p62 proteins is a well-known feature of autophagy-deficient tissues [32, 64, 80]. Levels of LC3-II, LC3-II/LC3-I and p62 were evaluated in hippocampal extracts from 5-month-old WT, G3Terc^−/−^, 5xFAD, and G3Terc^−/−^ 5xFAD mice (Fig. 7a). We observed a trend towards increased LC3-II, LC3-II/LC3-I and p62 levels in G3Terc^−/−^ mice. Consistent with previous reports [50, 118], further accumulation of LC3-II and p62 was observed in 5xFAD mice when compared to WT mice. This accumulation became significant in G3Terc^−/−^ 5xFAD mice, which could suggest an additive effect of both amyloid pathology and telomere attrition on autophagy impairment. Since this effect could be the result of either increased autophagic flux or a defective autophagy completion, we analyzed the levels of several proteins involved in the initiation stage of autophagy. Levels of the autophagy initiation proteins Atg9A and Beclin-1 were not altered in G3Terc^−/−^ 5xFAD mice when compared to WT mice (Fig. 7b), likely indicating that the observed autophagy impairment is the result of impaired clearance of autophagic vesicles. In addition, immunostaining analyses showed that p62 and LC3 partially colocalized with Aβ (Fig. 7c), suggesting that autophagosomes might be a reservoir of intracellular Aβ. However, it is important to be cautious with the interpretation of the LC3 staining, as studies previously demonstrated that LC3-positive punctate dots do not always represent autophagic structures, particularly in cells presenting protein aggregates [67]. In addition, the static evaluation of autophagy-related proteins, and particularly LC3-II, is not always a reliable method to determine autophagy alterations [63]. Thus, we aimed to further characterize the nature of the autophagic alteration caused by cellular senescence and evaluate its role in amyloid pathology. Autophagy is a complex and highly dynamic process, and therefore its evaluation in living cells often requires the use of an autophagy inhibitor, such as chloroquine, which decreases autophagosome-lysosome fusion and leads to the accumulation of autophagy-related proteins (Fig. 8a). The difference in the levels of these autophagy markers before and after treatment with chloroquine correlates with the number of autophagosomes that are being degraded upon fusion with the lysosome during a specific time frame [119]. We measured the state of the autophagic flux in primary neuronal cultures derived from WT and G3Terc^−/−^ mice by analyzing the levels of LC3 and p62 after chloroquine addition (Fig. 7b). WB analysis revealed that the accumulation of LC3-II/LC3-I and p62 levels after chloroquine treatment is decreased in G3Terc^−/−^ neurons compared to WT neurons (a non-significant trend for LC3-II/LC3 and a significant difference for p62 is observed), suggesting a reduction in autophagic flux (Fig. 8c). To investigate the role of the observed autophagy impairment on intracellular Aβ levels, we evaluated if autophagy activation could reverse intraneuronal Aβ accumulation in G3Terc^−/−^ neurons. WT and G3Terc^−/−^ primary neurons overexpressing hAPP3xmut (by lentiviral transduction, see Fig. 3) were treated for 4 days with either vehicle or the autophagy activator rapamycin. Autophagy activation by rapamycin was evidenced by decreased levels of p62 (Additional file 1: Fig. S7). Fluorescence quantification was then performed for Aβ42 immunostainings. Two-way ANOVA analyses revealed a significant effect of the genotype (F_1, 14_ = 9.835, *P* = 0.0073), indicating increased intraneuronal Aβ42 levels in G3Terc^−/−^ neurons (Fig. 8d). Strikingly, our results showed that treatment with rapamycin significantly decreased aberrant intraneuronal Aβ42 accumulation in both WT and G3Terc^−/−^ primary neurons. Human Aβ40 and Aβ42 levels were further quantified in those neurons using the ECLIA assays. Similarly, we found that the G3Terc^−/−^ genotype increased intraneuronal levels of both Aβ isoforms, while rapamycin treatment significantly reduced their accumulation (Fig. 8e), as Two-way ANOVA analyses detected significant effects based on genotype (for Aβ40, F_1, 10_ = 13.44, *P* = 0.0043; for Aβ42, F_1, 10_ = 9.891, *P* = 0.0104) and treatment (for Aβ40, F_1, 10_ = 5.615, *P* = 0.0393; for Aβ42, F_1, 10_ = 8.360, *P* = 0.0161). Taken together, our results indicate that senescent neurons display impaired autophagy, which might be involved in an aberrant accumulation of intraneuronal Aβ42.

**Fig. 9:**
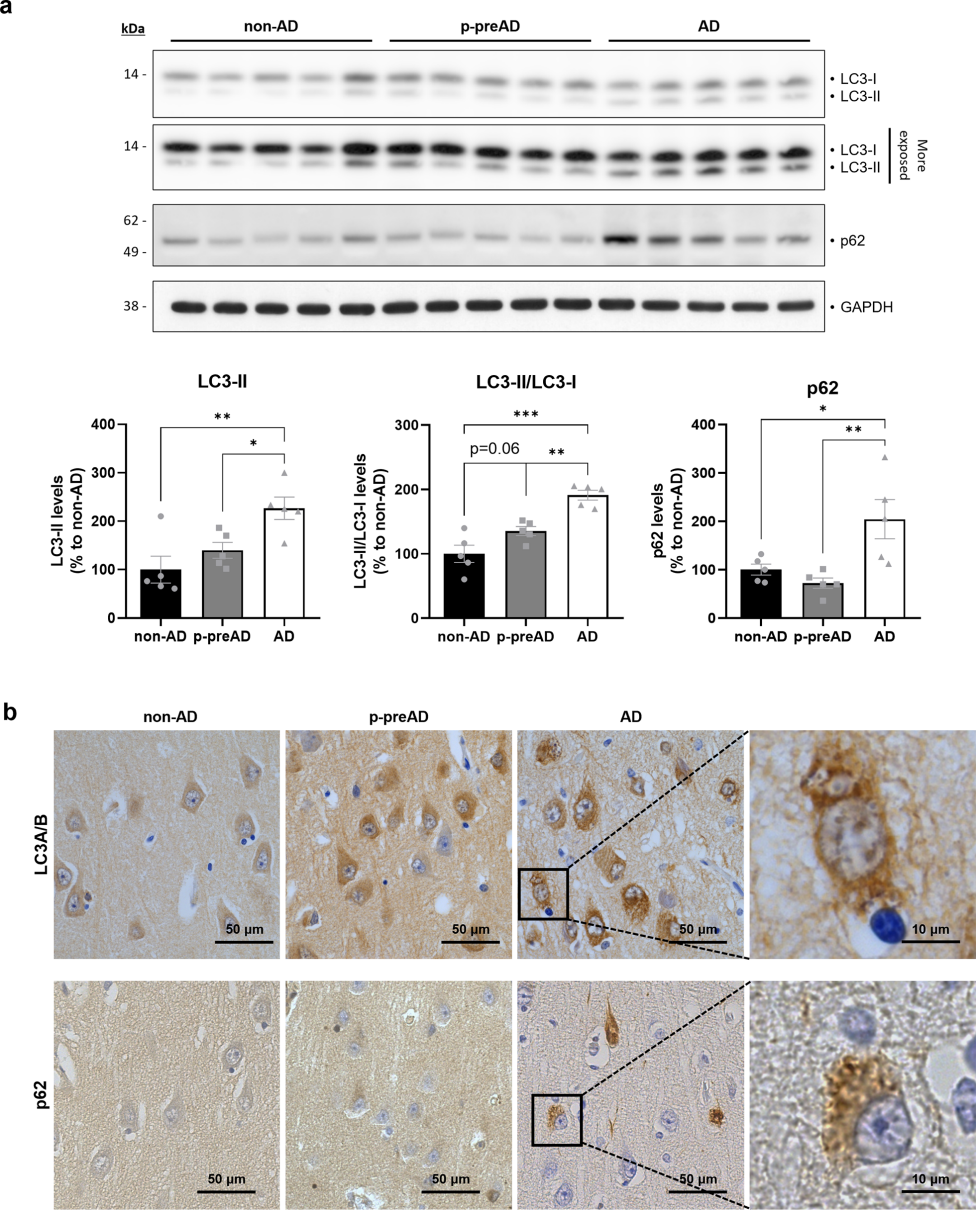
The brains of AD patients present altered autophagy. **a** Western blot analysis showing LC3 and p62 relative protein levels in the SDS-soluble fraction of homogenates derived from temporal cortex of non-AD, p-preAD and AD cases. GAPDH was used as loading control, and the levels in the control group were set as 100%. *P < 0.05, **P < 0.01, ***P<0.001 (One-way ANOVA with Tukey’s post-hoc analysis, n = 5 patients/group). Data are presented as mean ± SEM. **b** DAB immunostainings against LC3 and p62 in the CA1 hippocampal region of representative non-AD, p-preAD and AD cases. Scale bar: 50 μm or 10 μm (magnified image). non-AD: non-demented control; p-preAD: pathologically defined preclinical AD; AD: symptomatic AD

Next, we investigated if a similar autophagy impairment, reflected by increased LC3-II and p62 levels, was also observed in human AD brains. By performing WB analyses on postmortem human samples (Fig. 9a), we observed that LC3-II levels, the ratio of LC3-II to LC3-I, and p62 levels were significantly increased in AD brain samples when compared to non-AD controls. Additionally, there was a clear trend towards increased LC3-II and LC3-II/LC3-I levels in pathologically defined preclinical AD (p-preAD) cases, suggesting that autophagy alterations might appear early in the disease progression. We also performed LC3 and p62 immunostaining experiments on paraffin-embedded brain tissue sections from all three groups (Fig. 9b). LC3 staining was visually more abundant in hippocampal neurons of aged-matched p-preAD and AD patients compared to non-AD controls. For p62, we observed very low expression in control samples, but a certain degree of accumulation in some p-preAD cases. In AD cases, we detected the presence of neurons with strong p62 staining in the soma, with some intracellular inclusions highly resembling neurofibrillary tangles. WB analyses on postmortem human samples correlate those results with a significant increase in a phosphorylated form of the histone variant H2AX (γ-H2AX), a commonly used marker of cell senescence that indicates double-stranded DNA breaks [94] (Additional file 1: Fig. S8).

Overall, our results indicate the presence of an early autophagy alteration in AD human pathology.

## Discussion

**Fig. 10:**
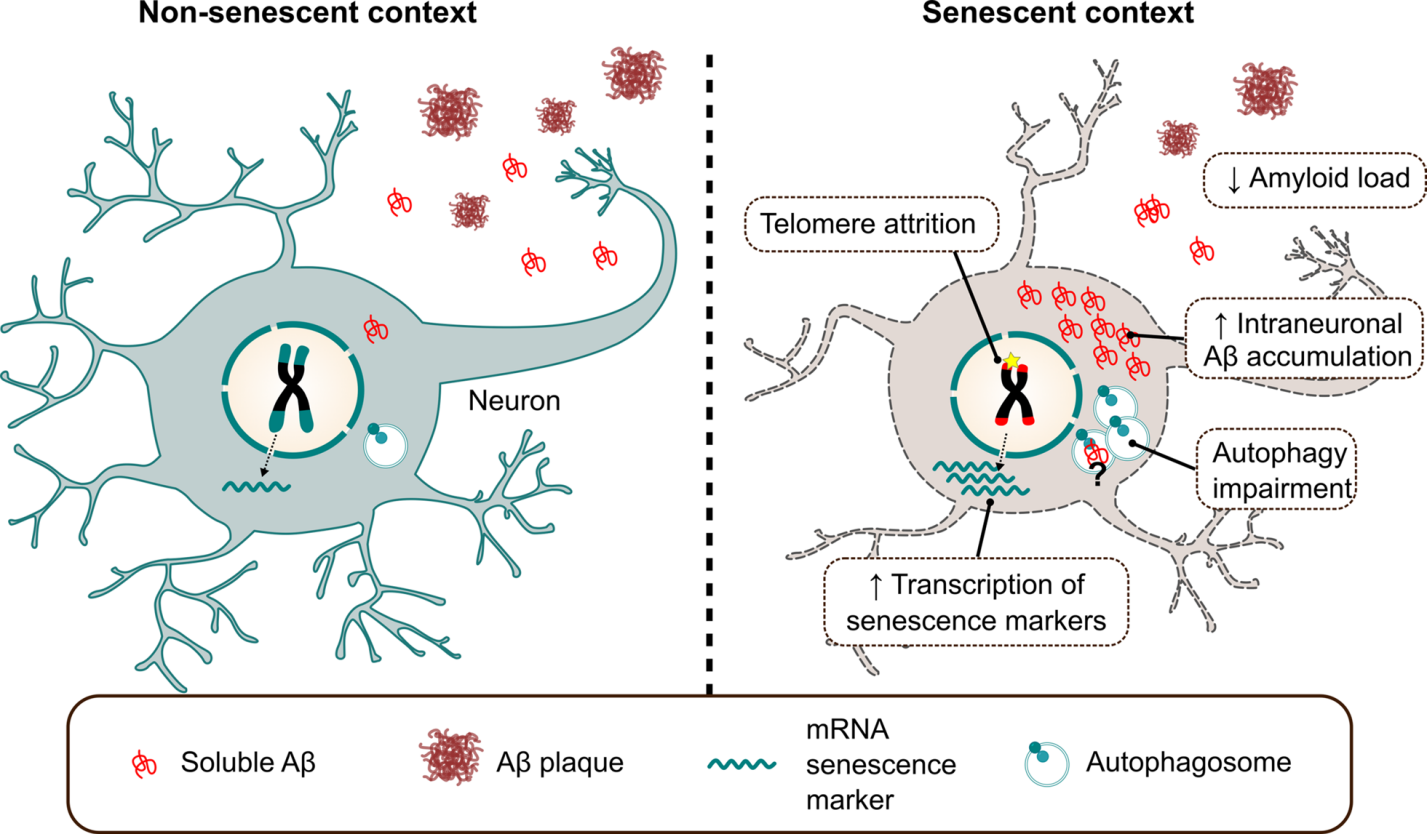
Graphical abstract summarizing the main findings of this article. In a senescent context that could be triggered by telomere attrition, neurons increase the transcription of classical senescence markers and display a diminished autophagic flux. Those alterations lead to an aberrant and neurotoxic increase in intracellular Aβ levels. Such an increase is later on translated into decreased extracellular Aβ plaque formation in connecting brain regions

Although compelling evidence puts aging as the main driver of AD and other neurodegenerative diseases, the exact mechanisms underlying pathological aging and how they relate to a specific neurodegenerative condition remain largely unknown. Senescence, as a fundamental aging process, has been recently investigated in aged and diseased brains, but its direct causative role in specific neurodegenerative pathways needs to be uncovered [114]. Telomere shortening is one of the most remarkable cellular alterations triggering senescence and it is considered to contribute to age-related diseases [97]. In this study, we provide first evidence that telomere-induced senescence enhances early intraneuronal Aβ accumulation and causes neuronal loss in specific brain areas of FAD mice, while reducing Aβ deposition at later disease stages. The main results of our study are summarized in Fig. 10. We propose that a disruption of the autophagy flux caused by cellular senescence might underlie those changes. A similar pattern of autophagic marker alteration is observed in vulnerable brain regions of AD patients.

We used here Terc-deficient mice as a mouse model of cellular senescence. We observed that the brains and primary neurons from G3Terc^−/−^ mice display telomere attrition and features of cellular senescence. Clear evidence for an effect of aging on telomere shortening in the brain, and particularly in postmitotic cells like neurons, has not been reported so far. Still, it has been shown that telomeres of various non-proliferative tissues, including the brain, are particularly resistant to DNA repair and can undergo persistent DNA damage response (DDR) even without being critically short [35]. Since neurons are terminally differentiated cells with high and continuous energy requirements and a predominant use of oxidative phosphorylation (OXPHOS) as energy supply, they are prone to accumulate cellular damage due to oxidative stress over extended periods of time. Identification of neuron-specific senescence markers and the characterization of neuronal damage associated with telomere attrition during aging would be of particular interest. Our results here extend previous knowledge on neuronal senescence [59] and validate the use of G3Terc^−/−^ mice to study the impact of this process on AD-related amyloid pathology.

The accumulation of the Aβ peptide is an early event that initiates AD pathophysiology [42, 45, 46]. Insoluble extracellular deposits made up of the Aβ peptide were initially discovered in the brains of all AD patients and are considered as a defining pathological hallmark, but their pathological properties seem restricted. It is now strongly hypothesized that other forms of Aβ (i.e., not the extracellular amyloid deposits) are underlying AD neurotoxicity. Supporting this idea, studies have shown that extracellular amyloid deposition correlates poorly with the degree of cognitive decline in AD [10], in agreement with the results obtained in clinical trials showing that Aβ-targeted immunotherapies removing Aβ deposition in the brain do not result in cognitive improvement [99]. Indeed, it has been repeatedly demonstrated that Aβ starts accumulating inside neurons and causing neuronal damage before it builds up in the extracellular space. Intraneuronal Aβ accumulation has been found in the brains of patients presenting AD or mild cognitive impairment [3, 21, 41, 47, 78], and studies suggest that this phenomenon represents an early step in AD neuropathology [40]. However, very little is known about the underlying mechanisms that cause elevated Aβ levels within neurons and how senescence-associated pathways could control intraneuronal Aβ accumulation. Our results indicate that accelerated senescence aggravates intraneuronal Aβ accumulation associated with neuronal loss. In line with our data, a very recent study has shown that *Tert* transcriptional activation in transgenic 3xTg-AD mice (which carry two FAD mutations together with a mutation of the *MAPT* gene [88]) reduces abnormal intracellular Aβ staining in the CA1 region and upregulates genes involved in synaptic signaling and neuronal survival [106]. Results from our study indicate for the first time that cellular senescence might increase intraneuronal Aβ by compromising autophagy function. The role of autophagy on intraneuronal Aβ is supported by a previous study showing that an AD-related amyloid mouse model (APP23^+^) presenting dysfunctional autophagy due to conditional knockout of Atg7 exhibits a substantial reduction of amyloid plaques but increased intraneuronal Aβ early in the pathology, which might trigger neurodegeneration and memory impairment [85]. The authors hypothesized that autophagy plays a key role in the balance between Aβ secretion and intracellular accumulation. Another piece of evidence for the role of autophagy in intraneuronal Aβ accumulation is the finding that intraneuronal Aβ correlates with the apolipoprotein E4 (ApoE4) genotype [21], the main known genetic risk factor for AD, which has been described to downregulate autophagy [108]. Interestingly, autophagy has been found altered in the liver of the G3Terc^−/−^ model [20] and a previous study found hyperactivation of the mTOR pathway, which is critically involved in autophagy inhibition, in the liver, skeletal muscle and heart of G2Terc^−/−^ mice [34]. In our study, we have shown that G3Terc^−/−^ primary neurons present reduced autophagy flux and increased Aβ accumulation, which can be reversed by activating autophagy using rapamycin, an mTOR inhibitor. In this regard, it is important to note that autophagy-inducing agents such as rapamycin have been used in preclinical and clinical trials for the treatment of AD with promising outcomes [104]. Additionally, we have shown that brain senescence causes autophagy deficiency in the brains of 5xFAD mice, in which we observed that Aβ partially colocalizes with autophagosomes, supporting the hypothesis that these biological structures are a reservoir of intracellular Aβ [120]. Our results on human brain samples provide further evidence that an autophagy dysfunction is found in the brains of AD patients, starting from early disease stages. A similar autophagy impairment has been previously found in brain tissues from AD patients, but data are controversial as they either support an alteration in autophagy induction or in the clearance of autophagosomes [75, 86]. A limitation of our study is the lack of intracellular Aβ evaluation in AD samples. Therefore, the extrapolation of our results obtained in mouse models to human pathology must be done with caution. Although compelling evidence of the intracellular Aβ phenomenon in AD has been consistently obtained over the last two decades [3, 21, 41, 47, 78], its evaluation in human post-mortem samples from AD patients is very complex due to several factors. On one hand, the presence of amyloid pathology in AD samples (mandatory for AD diagnosis) could mask the appearance of this feature. Indeed, intracellular Aβ accumulation in neurons that are particularly vulnerable in AD precedes plaque formation but decreases later on with the progression of Aβ deposition in both mice [89, 117] and humans [41, 81]. On the other hand, contrary to the classical AD-related mouse models of amyloid pathology, human AD brains do not overexpress APP and intracellular Aβ levels, though readily present, are expected to be very low. Additionally, Aβ staining could colocalize with or nonspecifically bind to lipofuscin [33, 38], and abnormal increases in lipofuscin are found in AD [29]. Conversely, other authors suggest that although there is some degree of colocalization, lipofuscin and Aβ42 might occupy distinct cellular compartments [26]. Long postmortem intervals could also be another confounding factor in the evaluation of this feature in human material, as compared to its detection in mice, in which samples are collected and rapidly fixed by transcardial perfusion in a well-controlled manner [6]. Finally, intracellular Aβ accumulation could also appear in non-AD controls as this could reflect early stages of AD pathology (especially in aged non-AD controls). Further studies should provide more insights into the mechanisms of autophagy dysregulation in AD and its role in intracellular Aβ accumulation, the relevance of which has been demonstrated in our study.

The underlying cause for the selective and early vulnerability of particular neurons in AD, as it happens for other neuronal populations in other neurodegenerative conditions, remains elusive. We showed that early intraneuronal Aβ42 immunoreactivity appears first in the subiculum (at 1.5 months of age) and later on in the cortical layer V (at 2 months of age) of 5xFAD mice, which is consistent with previous studies on AD mouse models [30, 87]. This feature correlates with the observation of reduced neuronal density in these two regions. While the evaluation of early AD-related alterations in the human brain is challenging, pathological studies have also indicated that the subiculum is one of the earliest affected regions in AD or prodromal AD patients [4, 15, 44, 73]. Conversely, we noticed that the 5xFAD model does not display intracellular Aβ42 immunoreactivity in any region of the proper hippocampus at any of the time points evaluated (1.5, 2 and 5 months), consistent with previous data [52, 57]. The lack of intraneuronal Aβ correlates with a complete preservation of neuronal density in the CA1, CA3 and DG regions of 5-month-old 5xFAD mice, despite amyloid plaques being abundant. This further supports the notion that the critical feature in Aβ-related neurotoxicity is intraneuronal accumulation rather than extracellular deposition. This idea is confirmed by studies reporting a transgenic mouse model bearing APP with a recently described AD-associated mutation that exhibits intraneuronal Aβ accumulation in the absence of plaques and still shows signs of AD-related neurodegeneration [113]. In contrast to 5xFAD, other FAD mouse models show clear intraneuronal Aβ accumulation and neuronal loss in the CA1 region [7, 16, 88], which has also been shown to be one of the earliest and more affected brain regions in AD patients [4, 27, 68, 82]. The differences observed between FAD mouse models in the initial site of intraneuronal Aβ might reflect differences in the promoter driving the expression of the transgene or in the site of insertion of the transgene. Further studies are needed to address how the different AD-related mouse models, including the recently generated knock-in models, correlate to the human pathology regarding the precise sub-regions of early intraneuronal Aβ accumulation.

A previous study demonstrated that crossing the Terc^−/−^ model with APP23 mice (which express human APP bearing the Swedish mutation [109]) causes an unexpected reduction in Aβ deposition [95], which the authors linked to telomere-dependent effects on microglia activation. Consistently, we observed that telomere attrition causes a similar decrease in amyloid deposition in 5xFAD mice. Our results show that APP expression, Aβ production, and glial activation are not modified by the senescent context. While astrocytes have been previously reported to be unaltered in G3Terc^−/−^ mice [95, 103, 116], contradictory results have been found on the activation of microglia in the brain of those mice [60, 91, 95, 103]. Our results indicate that amyloid pathology induces activation of astrocytes and microglia in 5xFAD mice, but the absence of further glial activation in G3Terc^−/−^ 5xFAD mice, while accompanied by a clear decrease in the number of Aβ plaques, led us to hypothesize that those cells are not directly responsible for the reduction in amyloid burden. Instead, our data suggest that the reduction in amyloid burden is a consequence of the early accumulation of intraneuronal Aβ in the accelerated senescence context. Indeed, there is a dynamic correlation between extra- and intracellular pools of Aβ, the latter being the source for some of the extracellular Aβ plaques [89]. However, while intraneuronal Aβ is observed in the subiculum, the reduction of amyloid burden was observed in different but anatomically interconnected brain regions, such as the cortex and hippocampus. Some studies have indicated that amyloid pathology might be gradually transmitted through connected brain structures [10, 69, 84, 96], by mechanisms that are still under investigation. Supporting the importance of the subiculum in the spread of AD pathology, a study showed that lesioning the dorsal subiculum of a mouse model of AD amyloidosis reduces the spread of amyloid pathology in brain regions projecting to and receiving connections from this region, such as the CA1 and retrosplenial cortex [37]. The authors suggest that this might be the result of an alteration in the delivery of APP and/or APP fragments from the subiculum to connecting regions. Further knowledge on the biological mechanisms that regulate the equilibrium between intra- and extracellular pools of Aβ and their role in the spreading of amyloid pathology is much needed.

## Conclusions

Overall, our data demonstrated that accelerated senescence induces neurotoxic intraneuronal Aβ accumulation, probably through autophagy dysfunction, shedding light on the causative role of pathological aging in AD. Further experiments should be performed to decipher the specific molecular pathways that are affected during senescence and that modulate Aβ-induced neurotoxicity, which could set the basis for testing novel and promising targets suitable for pharmacological intervention in AD.

## Supplementary Information

**Additional file 1. **This file includes supplementary Figures S1–S8, supplementary Methods and supplementary Tables S1–S6.

## Data Availability

All datasets generated and analyzed during this study are included in this published article and its supplementary material.

## Abbreviations

AD: Alzheimer's disease
ApoE: Apolipoprotein E
APP: Amyloid precursor protein
Aβ: Amyloid β
CTF: C-terminal fragment of APP
ECLIA: Electrochemiluminescence immunoassay
FAD: Familial Alzheimer's disease
non-AD: Non-demented control
p-preAD: Pathologically defined preclinical AD
sAPP: Soluble APP
SASP: Senescence-associated secretory phenotype
SA-β-gal: Senescence-associated β-galactosidase
ThT: Thioflavin T
WB: Western blot
WT: Wild-type
