# Supplementary material for: Senescence-related impairment of autophagy induces toxic intraneuronal amyloid-β accumulation in a mouse model of amyloid pathology
Source: Acta Neuropathol Commun. 2023 May 17;11:82. doi: 10.1186/s40478-023-01578-x (PMC10189946; doi:10.1186/s40478-023-01578-x)
Supplement: Supplementary file 1 — Additional file 1. This file includes supplementary Figures S1–S8, supplementary Methods and supplementary Tables S1–S6. [file 40478_2023_1578_MOESM1_ESM.docx]

Supplementary Information

Senescence-related impairment of autophagy induces toxic intraneuronal amyloid-β accumulation in a mouse model of amyloid pathology.

Nuria Suelves^1^, Shirine Saleki^1^, Tasha Ibrahim^1,#^, Debora Palomares^1,#^, Sebastiaan Moonen^2,3,4^, Marta J Koper^2,3,4^, Céline Vrancx^1,5^, Devkee M Vadukul^1,6^, Nicolas Papadopoulos^7,8^, Nikenza Viceconte^9,10^, Eloïse Claude^9^, Rik Vandenberghe^11,12^, Christine AF von Arnim^13,14^, Stefan N Constantinescu^7,8,15,16^, Dietmar Rudolf Thal^2,17^, Anabelle Decottignies^9^, Pascal Kienlen-Campard^1&^

^1^Aging and Dementia Group, Cellular and Molecular Division (CEMO), Institute of Neuroscience (IoNS), UCLouvain, Brussels, Belgium.

^2^Laboratory for Neuropathology, Department of Imaging and Pathology, Leuven Brain Institute (LBI), KU Leuven, Leuven, Belgium.

^3^Laboratory for the Research of Neurodegenerative Diseases, Department of Neurosciences, Leuven Brain Institute (LBI), KU Leuven, Leuven, Belgium.

^4^Vlaams Instituut voor Biotechnologie (VIB) Center for Brain and Disease Research, VIB, Leuven, Belgium.

^5^*Current affiliation:* Laboratory for Membrane Trafficking, Department of Neurosciences, Vlaams Instituut voor Biotechnologie (VIB) Center for Brain and Disease Research, KU Leuven, Leuven, Belgium.

^6^*Current affiliation:* Department of Chemistry, Molecular Sciences Research Hub, Imperial College London, London, United Kingdom.

^7^Ludwig Institute for Cancer Research, Brussels, Belgium.

^8^SIGN Unit, de Duve Institute, UCLouvain, Brussels, Belgium.

^9^Genetic & Epigenetic Alterations of Genomes Unit, de Duve Institute, UCLouvain, Brussels, Belgium.

^10^*Current affiliation:* CENTOGENE GmbH, 18055, Rostock, Germany.

^11^Laboratory for Cognitive Neurology, Department of Neurosciences, Leuven Brain Institute (LBI), KU Leuven (University of Leuven), Leuven, Belgium.

^12^Department of Neurology, University Hospital Leuven, Leuven, Belgium.

^13^Department of Neurology, University of Ulm, Ulm, Germany.

^14^Department of Geriatrics, University Medical Center Göttingen, Göttingen, Germany.

^15^Walloon Excellence in Life Sciences and Biotechnology (WELBIO), Brussels, Belgium.

^16^Ludwig Institute for Cancer Research, Nuffield Department of Medicine, Oxford University, Oxford, UK.

^17^Department of Pathology, University Hospital Leuven, Leuven, Belgium.

^#^Contributed equally.

**^&^Corresponding author:** Pascal Kienlen-Campard. Email: [pascal.kienlen-campard@uclouvain.be](mailto:pascal.kienlen-campard@uclouvain.be). ORCID: [0000-0003-1086-2942](https://orcid.org/0000-0003-1086-2942)

# Supplementary Figures and Legends


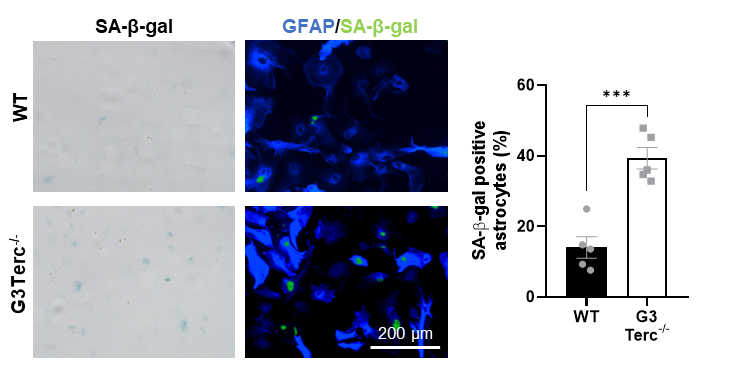


**Figure S1** Increased senescence-associated β-galactosidase (SA-β-gal) activity in primary astrocytes from G3Terc^-/-^ mice. Primary astrocytes obtained from WT and G3Terc^-/-^ mice were stained for SA-β-gal, followed by immunostaining using the selective astrocyte marker GFAP (blue), and the percentage of SA-β-gal-positive astrocytes was calculated. ****P* < 0.001 (two-tailed Student’s *t-*test, n = 5). All data are presented as the mean ± SEM. Scale bar: 200 μm.


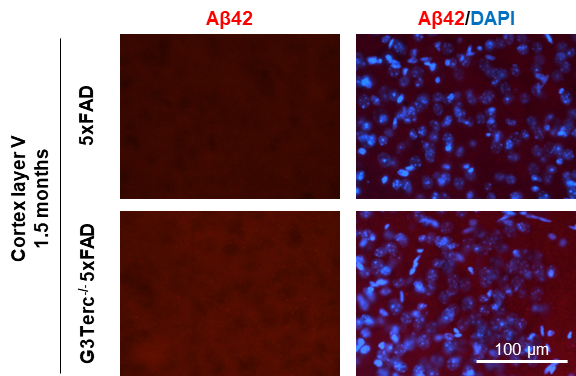


**Figure S2** Lack of intraneuronal Aβ accumulation in the cortical layer V region of 1.5-month-old 5xFAD mice. Immunostaining analysis of Aβ42 (Aβ42 antibody, clone H31L21, red) in the cortical layer V region from 1.5-month-old 5xFAD and G3Terc^-/-^ 5xFAD mice. Representative photomicrographs are shown. Scale bar: 100 μm.

**
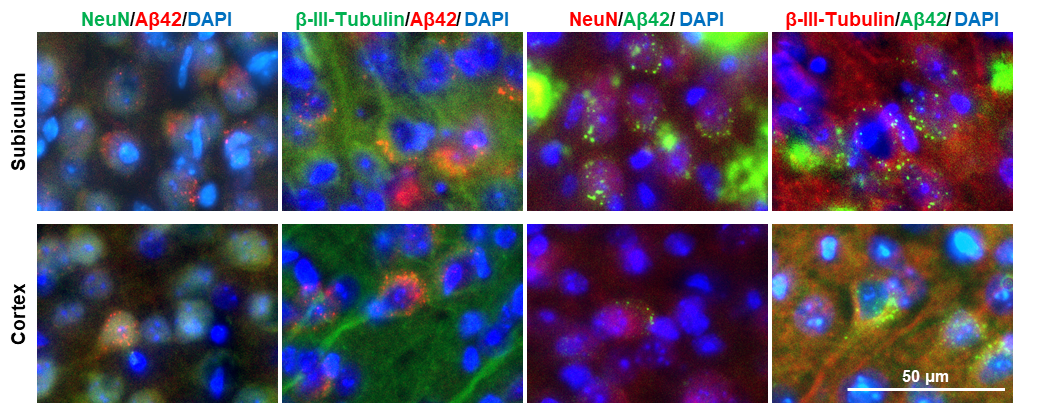
**

**Figure S3** Early Aβ accumulation in 5xFAD mice is intraneuronal. Colocalization of Aβ42 (clone H31L21, red or green) with two different neuronal markers (NeuN or β-III-Tubulin, red or green) in the subiculum (upper panel) or cortex (lower panel) of 2-months-old 5xFAD mice. Representative photomicrographs are shown. Scale bar: 50 μm.


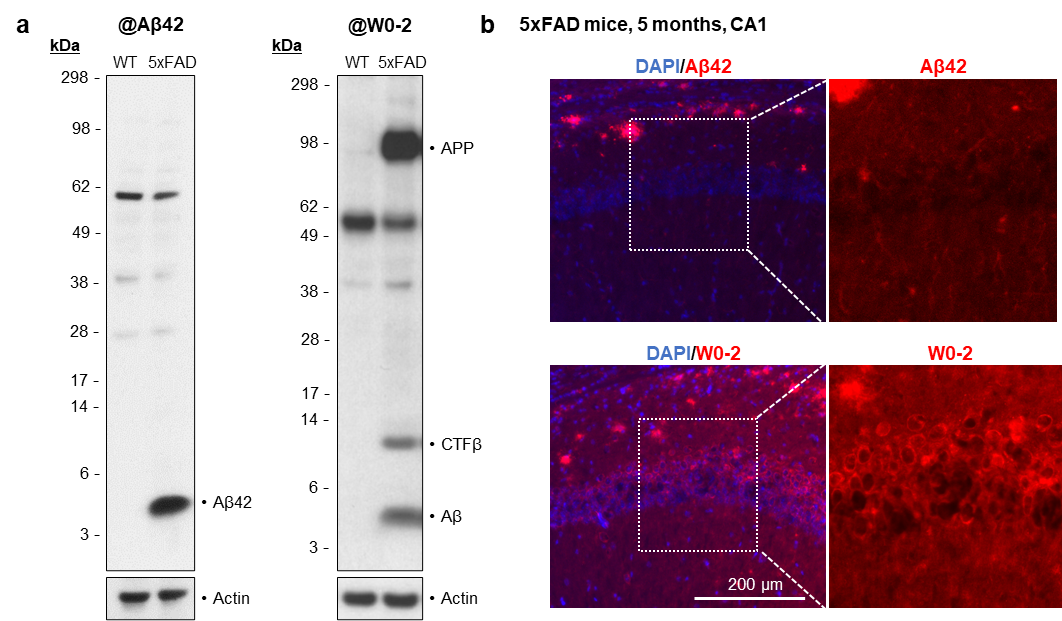


**Figure S4** Specificity of the Aβ42 antibody used in this study. **a**) Western blot analysis of C-terminal Aβ42 antibody (clone H31L21, left panel) and N-terminal Aβ antibody (clone W0-2, right panel) using hippocampal brain extracts from WT and 5xFAD mice at 5 months of age. Actin was used as loading control. Molecular Weight markers (in kDa) are shown. **b**) Characterization of intraneuronal staining for C-terminal Aβ42 antibody (clone H31L21, red, upper panel) and N-terminal Aβ antibody (clone W0-2, red, lower panel) in the CA1 region of 5xFAD mice at 5 months of age. Representative low and high magnification (inset derived from marked location on low-magnification images) photomicrographs are shown. Scale bar: 200 μm.

**
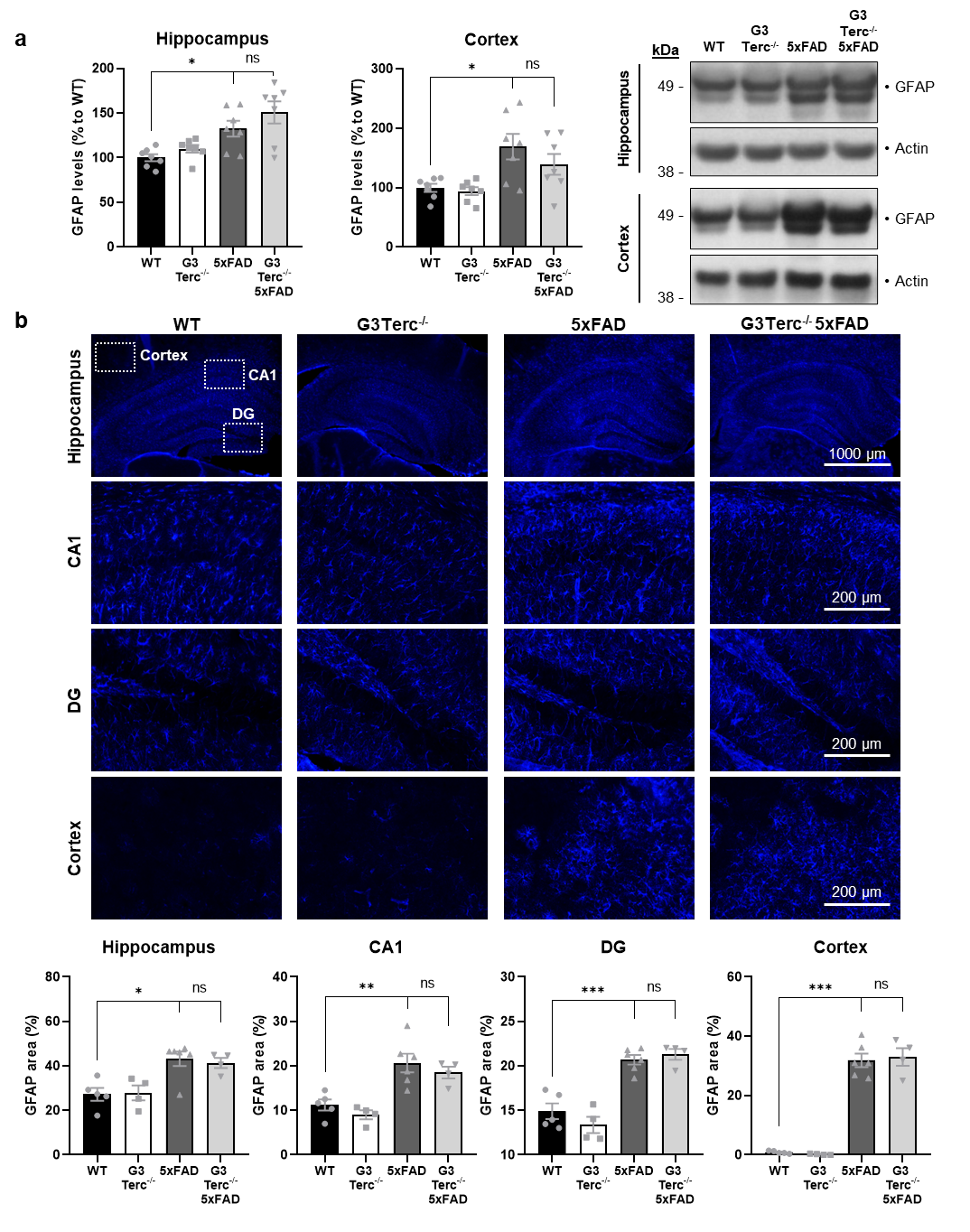
**

**Figure S5** Astrocytes are not further activated by telomere attrition in the context of amyloid pathology. **a**) Western blot analysis showing protein levels of GFAP in hippocampal and cortical protein extracts from 5-month-old WT, G3Terc^-/-^, 5xFAD and G3Terc^-/-^ 5xFAD mice. Actin was used as loading control, and the levels in the control group were set as 100%. **P* < 0.05 (One-way ANOVA with Tukey’s post-hoc analysis, n = 7). **b**) Immunostaining analysis of GFAP-positive astrocytes (blue) in 5-month-old WT, G3Terc^-/-^, 5xFAD and G3Terc^-/-^ 5xFAD brains. Representative photomicrographs are shown for each genotype in selected brain regions. Scale bar: 1000 μm (hippocampus) or 200 μm (CA1, DG, cortex). Quantitative analysis of astrocyte activation was performed by measuring the area (%) covered by GFAP staining. **P* < 0.05, ***P* < 0.01, ****P* < 0.001 (One-way ANOVA with Tukey’s post-hoc analysis, n = 4-6). All data are presented as the mean ± SEM.


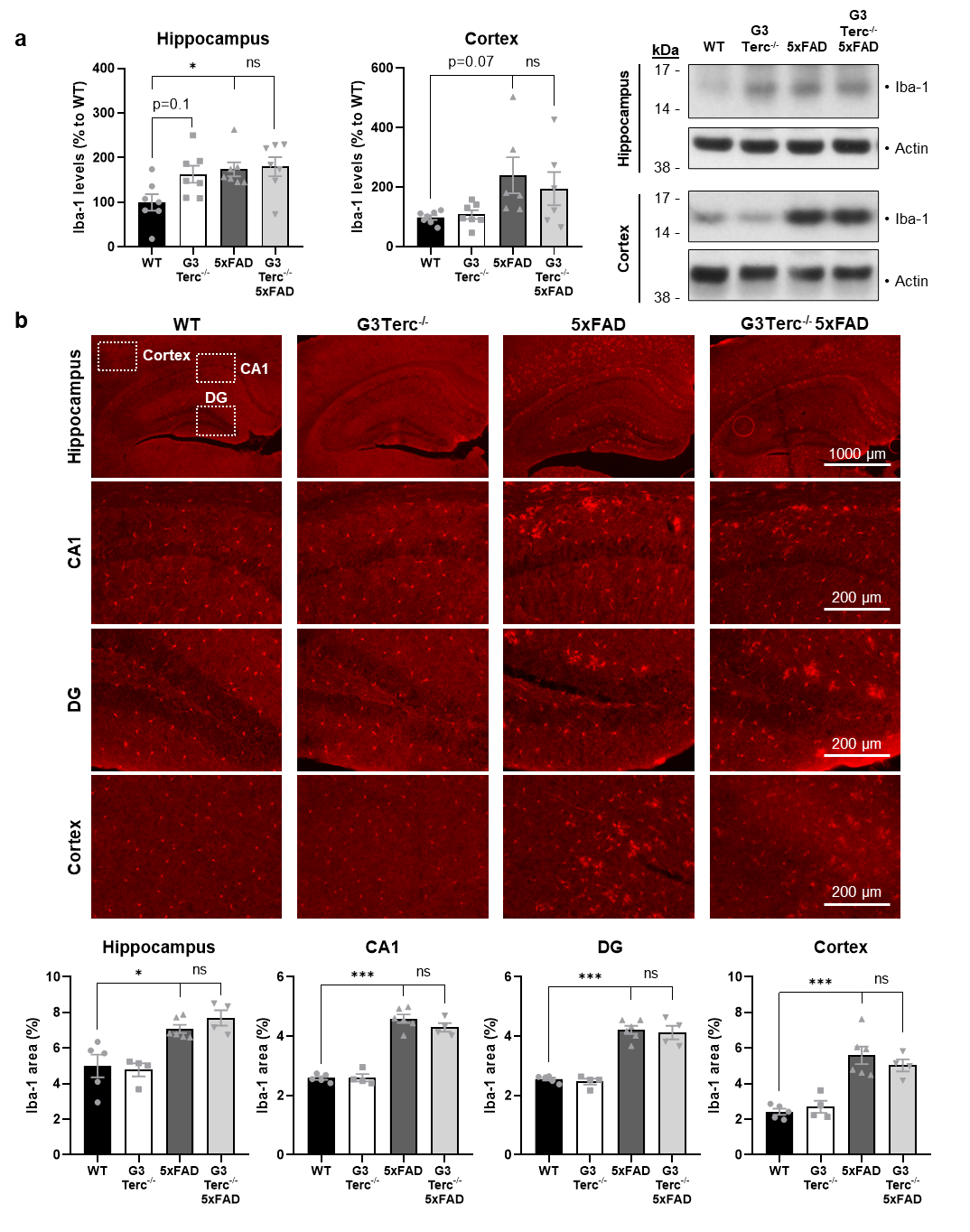


**Figure S6** Microglial cells are not further activated by telomere attrition in the context of amyloid pathology. **a**) Western blot analysis showing protein levels of Iba-1 in hippocampal and cortical protein extracts from 5-month-old WT, G3Terc^-/-^, 5xFAD and G3Terc^-/-^ 5xFAD mice. Actin was used as loading control, and the levels in the control group were set as 100%. **P* < 0.05 (One-way ANOVA with Tukey’s post-hoc analysis, n = 7). **b**) Immunostaining analysis of Iba-1-positive microglia (red) in 5-month-old WT, G3Terc^-/-^, 5xFAD and G3Terc^-/-^ 5xFAD brains. Representative photomicrographs are shown for each genotype in selected brain regions. Scale bar: 1000 μm (hippocampus) or 200 μm (CA1, DG, cortex). Quantitative analysis of microglia activation was performed by measuring the area (%) covered by Iba-1 staining. **P* < 0.05, ****P* < 0.001 (One-way ANOVA with Tukey’s post-hoc analysis, n = 4-6). All data are presented as the mean ± SEM.

**
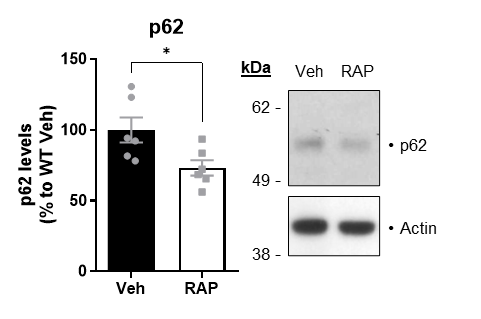
**

**Figure S7** Autophagy activation by Rapamycin in primary neurons. Western blot analysis showing p62 protein levels in WT primary neurons treated with rapamycin (RAP) or vehicle (Veh) for 24h or 4 days. Actin was used as loading control, and the levels in the control group were set as 100%. **P* < 0.05 (two-tailed Student’s *t-*test, n= 6). All data are presented as mean ± SEM.


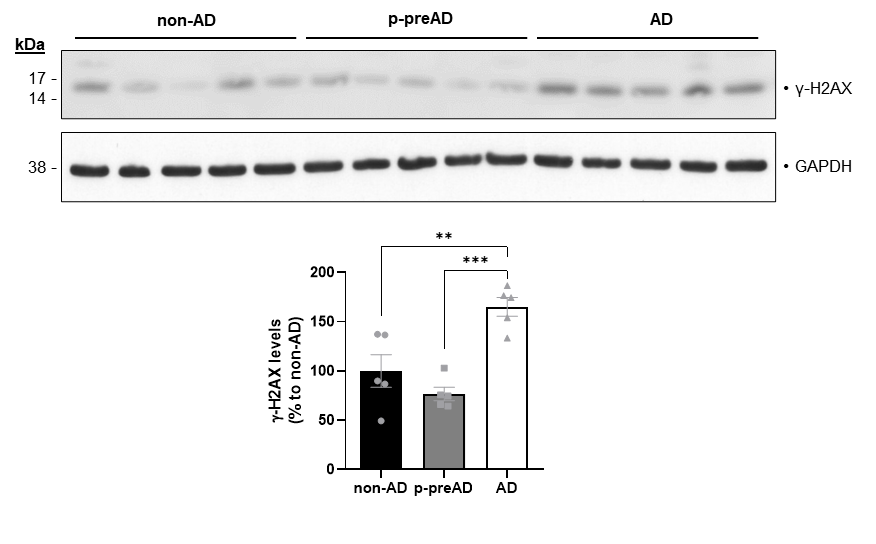


**Figure S8** Senescence activation in the brains of AD patients. Western blot analysis showing γ-H2AX relative protein levels in the SDS-soluble fraction of homogenates derived from temporal cortex of non-AD, p-preAD and AD cases. GAPDH was used as loading control, and the levels in the control group were set as 100%. ***P* < 0.01, ****P* < 0.001 (One-way ANOVA with Tukey’s post-hoc analysis, n = 5 patients/group). All data are presented as mean ± SEM.

# Supplementary Methods

## Primary astrocyte cultures

## Primary astrocyte cultures were obtained from mouse pups aged 2 days as previously described [1]. Briefly, cortices were isolated on ice-cold HBSS and dissociated by sequentially using a glass pipette and a flame-narrowed glass pipette. Samples were centrifuged 1000×g for 5 min. Pellets were resuspended in HBSS and centrifuged at 1,700 × g for 20 min on a 30% Percoll gradient. Astrocytes were collected from the interphase, washed in HBSS and centrifuged for 5 min at 1,500 × g. Pellets were resuspended and plated in DMEM-glutaMAX medium (Thermo Fisher Scientific) supplemented with 10% FBS (Biowest), 50 mg/ml penicillin–streptomycin, and 50 mg/ml fungizone. Cells were left to proliferate in flasks for 15 days at 37°C and 5% CO2, and media were changed every 4–5 days. After 15 days, astrocytes were plated and further cultured in DMEM-glutaMAX with 10% FBS. Two days later, differentiation was induced by reducing the concentration of FBS to 3% for 7 days before performing experiments.

## Senescence-associated β-galactosidase staining for astrocytes

The SA-β-gal staining was performed as described in the “Senescence-associated β-galactosidase (SA-β-gal) staining for neurons” section of “Materials and methods”. After staining, cells were then washed and processed for GFAP immunostaining as described in the “Immunofluorescence stainings” section of “Materials and methods”.

# Supplementary Tables

| Case number | Age | Sex | Aβ MTL phase | Braak NFT stage | CERAD score | NIA-AA score | CDR score | pMLKL-GVD  stage | Neuropathological diagnosis | PMI |
| --- | --- | --- | --- | --- | --- | --- | --- | --- | --- | --- |
| 1** | 87 | m | 4 | 6 | 2 | 3 | 2 | 5 | AD | 12 |
| 2* | 69 | m | 4 | 6 | 2 | 3 | 1 | 5 | AD | 24 |
| 3** | 57 | m | 4 | 6 | 3 | 3 | 3 | 5 | AD | 12 |
| 4* | 55 | f | 4 | 6 | 3 | 3 | 3 | 5 | AD | 24 |
| 5 | 74 | f | 4 | 6 | 2 | 3 | n.d. | 5 | AD | 72 |
| 6 | 71 | f | 4 | 6 | 2 | 3 | 3 | 5 | AD | 24 |
| 7** | 76 | m | 4 | 5 | 3 | 3 | 3 | 5 | AD | 24 |
| 8 | 78 | f | 4 | 5 | 2 | 3 | 0 | 4 | AD | 48 |
| 9 | 78 | f | 3 | 5 | 3 | 3 | 1 | 5 | AD, B | 10 |
| 10 | 89 | f | 4 | 4 | 3 | 2 | 0 | 4 | AD, CAA | 14 |
| 11 | 82 | m | 2 | 4 | 2 | 2 | 3 | 5 | AD | 24 |
| 12 | 82 | m | 3 | 4 | 2 | 2 | 0 | 5 | AD | n.d. |
| 13 | 87 | f | 4 | 3 | 1 | 2 | 0 | 4 | AD, CAA, B | n.d. |
| 14* | 74 | m | 4 | 3 | 1 | 2 | 0 | 4 | p-preAD | 48 |
| 15 | 74 | m | 4 | 3 | 0 | 2 | n.d. | 3 | p-preAD | 96 |
| 16 | 84 | f | 3 | 3 | 0 | 2 | 0 | 3 | p-preAD, I | 96 |
| 17 | 62 | m | 4 | 3 | 0 | 2 | 0 | 4 | p-preAD | 23 |
| 18 | 72 | m | 2 | 3 | 0 | 1 | 0 | 4 | p-preAD, ARTAG, I | n.d. |
| 19** | 89 | m | 3 | 2 | 0 | 1 | 0 | n.d. | p-preAD, MI | n.d. |
| 20 | 83 | f | 3 | 2 | 1 | 2 | 0 | 4 | p-preAD, AGD | n.d. |
| 21 | 81 | m | 3 | 2 | 0 | 1 | 0 | 0 | p-preAD, AGD, ADRP | 9 |
| 22** | 77 | f | 3 | 2 | 0 | 1 | 0 | 2 | p-preAD, I | 48 |
| 23 | 67 | m | 1 | 2 | 0 | 1 | 0 | 2 | p-preAD | 0 |
| 24* | 67 | f | 2 | 2 | 0 | 1 | n.d. | 2 | p-preAD | 96 |
| 25** | 73 | f | 1 | 2 | 0 | 1 | 0 | 1 | p-preAD | n.d. |
| 26 | 71 | m | 2 | 1 | 0 | 1 | 0 | 0 | p-preAD, CAA | 48 |
| 27 | 64 | m | 2 | 1 | 0 | 1 | 0 | 1 | p-preAD | 0 |
| 28* | 46 | m | 0 | 1 | 0 | 0 | 0 | 0 | Non-AD control | 29 |
| 29 | 69 | f | 0 | 0 | 0 | 0 | 0 | 0 | Non-AD control | 24 |
| 30 | 61 | m | 0 | 0 | 0 | 0 | 0 | 0 | Non-AD control, CM | 24 |
| 31 | 61 | m | 0 | 0 | 0 | 0 | 0 | 0 | Non-AD control | 24 |
| 32 | 32 | m | 0 | 0 | 0 | 0 | 0 | 0 | Non-AD control | n.d. |
| 33** | 64 | m | 0 | 0 | 0 | 0 | 0 | 0 | Non-AD control | n.d. |
| 34** | 67 | m | 0 | 0 | 0 | 0 | 0 | 0 | Non-AD control | n.d. |
| 35 | 60 | m | 0 | 0 | 0 | 0 | 0 | 1 | Non-AD control, I | n.d. |
| 36* | 74 | m | 0 | 0 | 0 | 0 | 0 | 0 | Non-AD control, CM, I, MI | 72 |
| 37 | 56 | m | 0 | 0 | 0 | 0 | 0 | 0 | Non-AD control | n.d. |
| 38* | 45 | m | 0 | 0 | 0 | 0 | 0 | 0 | Non-AD control | n.d. |
| 39 | 18 | m | 0 | 0 | 0 | 0 | 0 | 0 | Non-AD control, T | n.d. |

**Table S1** Overview of the human cohort characteristics

The table summarizes cohort characteristics, including age in years, sex, Aβ plaque pathology (Aβ MTL phase), NFT distribution in the brain (Braak NFT stage), the frequency of neuritic plaques (CERAD score), the degree of AD pathology determined using previously mentioned pathological parameters (NIA-AA score), the degree of dementia (CDR score), pMLKL-GVD stage, neuropathological diagnosis and PMI. Case numbers marked by a single asterisk were used in western blot and IHC experiments. Double asterisks indicate cases only used for western blot. Cases without asterisk were only used for IHC stainings. f: female; m: male; Aβ: amyloid β; AD: Alzheimer’s disease; p-preAD: pathologically defined preclinical AD; non-AD: non-demented control; ADRP: Alzheimer’s disease-related metabolic brain pattern; AGD: argyrophilic grain disease; ARTAG: aging-related tau astrogliopathy; B: bleeding; CAA: cerebrovascular angiopathy; CDR: clinical dementia rating; CERAD: Consortium to Establish a Registry for Alzheimer’s disease; CM: carcinoma metastasis; I: infarction; MI: microinfarction; MCI: mild cognitive impairment; MTL: medial temporal lobe; n.d.: not determined; NFT: neurofibrillary tangle; NIA-AA: National Institute on Aging–Alzheimer’s Association; PMI: post-mortem interval; T: trauma.

## Table S2 Sequences of primers used for telomere length analysis

| Target | Forward (5’- 3’) | Reverse (5’- 3’) |
| --- | --- | --- |
| Telomere | CGGTTTGTTTGGGTTTGGGT TTGGGTTTGGGTTTGGGTT | GGCTTGCCTTACCCTTACCC TTACCCTTACCCTTACCCT |
| *36b4* | ACTGGTCTAGGACCCGAGAAG | TCAATGGTGCCTCTGGAGATT |

## Table S3 Sequences of primers used for quantitative RT-PCR

| **Target** | **Forward (5’- 3’)** | **Reverse (5’- 3’)** |
| --- | --- | --- |
| ***Il1b*** | GCAACTGTTCCTGAACTCAACT | ATCTTTTGGGGTCCGTCAACT |
| ***Il6*** | TAGTCCTTCCTACCCCAATTTCC | TTGGTCCTTAGCCACTCCTTC |
| ***Cxcl1*** | AACCGAAGTCATAGCCACAC | GACACCTTTTAGCATCTTTTGG |
| ***P16*** | CCCAACGCCCCGAACT | GCAGAAGAGCTGCTACGTGAA |
| ***P19*** | CGCAGGTTCTTGGTCACTGT | TGTTCACAGAAGCCAGAGCG |
| ***P21*** | CCTGGTGATGTCCGACCTG | CCATGAGCGCATCGCAATC |
| **Human *APP*** | CAGCATTTCCAGGAGAAAGT | CTGCAGAGCGGTGATGTAGT |
| **Mouse *APP*** | AATGAGAGACAGCAGCTTGT | TGCAGTGCAGTGATGTAATT |
| ***Gapdh*** | ACCCAGAAGACTGTGGATGG | ACACATTGGGGGTAGGAAC |

## Table S4 Information on primary antibodies used on mouse samples

| **Target** | **Company** | **Application (dilution)** | **Reference** |
| --- | --- | --- | --- |
| **Actin** | Sigma-Aldrich | WB (1:1000) | A2066 |
| **APP** | Sigma-Aldrich | WB (1:2000) | A8717 |
| **Atg9A** | Abcam | WB (1:1000) | ab108338 |
| **Aβ, clone MOAB-2** | Biosensis | IHC-IF (1:200) | M-1586-100 |
| **Aβ human, clone W0-2** | Sigma-Aldrich | WB (1:1000), IHC-IF (1:100) | MABN10 |
| **Aβ42, clone H31L21** | Thermo Fisher Scientific | WB (1:1000), IHC-IF (1:200 [for intraneuronal staining], 1:500 [for plaque staining]), ICC-IF (1:100) | 700254 |
| **Beclin-1** | Cell Signaling Technology | WB (1:1000) | 3495T |
| **GFAP** | Thermo Fisher Scientific | WB (1:1000) | MA5-12023 |
| **GFAP** | Abcam | ICC-IF (1:100), IHC-IF (1:1000) | ab4674 |
| **Iba-1** | Abcam | WB (1:1000) | ab178846 |
| **LC3B** | Cell Signaling Technology | WB (1:1000), IHC-IF (1:100) | 3868S |
| **MAP2** | Sigma-Aldrich | ICC-IF (1:1000) | M4403 |
| **NeuN** | Abcam | IHC-IF (1:1000) | ab104225 |
| **NeuN** | Sigma-Aldrich | IHC-IF (1:200) | MAB377 |
| **Presenilin 1** | Cell Signaling Technology | WB (1:1000) | 5643S |
| **Presenilin 2** | Cell Signaling Technology | WB (1:1000) | 9979S |
| **p62** | Cell Signaling Technology | WB (1:1000), IHC-IF (1:50) | 23214S |
| **β-III-Tubulin** | Biolegend | IHC-IF (1:100) | 801213 |

## Table S5 Information on primary antibodies used on human samples

| **Target** | **Company** | **Application (dilution)** | **Reference** |
| --- | --- | --- | --- |
| **GAPDH** | Thermo Fisher Scientific | WB (1:10,000) | AM4300 |
| **LC3B** | Cell Signaling Technology | WB (1:1000) | 3868S |
| **LC3A/B** | Cell Signaling Technology | IHC-DAB (1:500) | 12741 |
| **p62** | BD Transduction Laboratories | WB (1:1000), IHC-DAB (1:250) | 610833 |
| **γ-H2AX** | Sigma-Aldrich | WB (1:1000) | 05-636 |

## Table S6 Fluorophore-conjugated secondary antibodies

| **Probe** | **Company** | **Reference** |
| --- | --- | --- |
| **Alexa Fluor 488 goat anti-mouse IgG (H+L)** | Invitrogen | A11029 |
| **Alexa Fluor 488 goat anti-rabbit IgG (H+L)** | Invitrogen | A11034 |
| **Alexa Fluor 568 goat anti-mouse IgG (H+L)** | Invitrogen | A11031 |
| **Alexa Fluor 568 goat anti-rabbit IgG (H+L)** | Invitrogen | A11036 |
| **Alexa Fluor 647 goat anti-chicken IgY (H+L)** | Invitrogen | A21449 |

# References

1 Contino S, Suelves N, Vrancx C, Vadukul DM, Payen VL, Stanga S et al (2020) Presenilin-Deficient Neurons and Astrocytes Display Normal Mitochondrial Phenotypes. Front Neurosci 14: 586108 Doi 10.3389/fnins.2020.586108
